# A Transcription Activator-Like Effector Tal7 of *Xanthomonas oryzae* pv. *oryzicola* Activates Rice Gene *Os09g29100* to Suppress Rice Immunity

**DOI:** 10.1038/s41598-017-04800-8

**Published:** 2017-07-11

**Authors:** Lulu Cai, Yanyan Cao, Zhengyin Xu, Wenxiu Ma, Muhammad Zakria, Lifang Zou, Zaiquan Cheng, Gongyou Chen

**Affiliations:** 1 School of Agriculture and Biology, Shanghai Jiao Tong University/Key Laboratory of Urban (South) by Ministry of Agriculture, Shanghai, 200240 China; 2 State Key Laboratory of Microbial Metabolism, School of Life Science & Biotechnology, Shanghai Jiao Tong University, Shanghai, 200240 China; 3 Biotechnology and Genetic Resource Research Institute, Yunnan Academy of Agricultural Sciences, Kunming, 650223 China

## Abstract

*Xanthomonas oryzae* pv. *oryzicola* (*Xoc*) and *X*. *oryzae* pv. *oryzae* (*Xoo*) cause bacterial leaf streak (BLS) and bacterial leaf blight (BLB) in rice, respectively. Unlike *Xoo*, endogenous avirulence-resistance (*avr*-*R*) gene interactions have not been identified in the *Xoc-*rice pathosystem; however, both pathogens possess transcription activator-like effectors (TALEs) that are known to modulate *R* or *S* genes in rice. The transfer of individual *tal* genes from *Xoc* RS105 (hypervirulent) into *Xoc* YNB0-17 (hypovirulent) led to the identification of *tal7*, which suppressed *avrXa7-Xa7* mediated defense in rice containing an *Xa7 R* gene. Mobility shift and microscale thermophoresis assays showed that Tal7 bound two EBE sites in the promoters of two rice genes, *Os09g29100* and *Os12g42970*, which encode predicted Cyclin-D4-1 and GATA zinc finger family protein, respectively. Assays using designer TALEs and a TALE-free strain of *Xoo* revealed that *Os09g29100* was the biologically relevant target of Tal7. Tal7 activates the expression of rice gene *Os09g29100* that suppresses *avrXa7-Xa7* mediated defense in Rice. TALEN editing of the Tal7-binding site in the *Os09g29100* gene promoter further enhanced resistance to the pathogen *Xoc* RS105. The suppression of effector-trigger immunity (ETI) is a phenomenon that may contribute to the scarcity of BLS resistant cultivars.

## Introduction

*Xanthomonas oryzae* pv. *oryzicola* (*Xoc*) causes bacterial leaf streak (BLS) in rice^1, 2^, a disease that is endemic in rice-growing regions worldwide. In China, BLS is more devastating than bacterial leaf blight (BLB) of rice caused by *X*. *oryzae* pv. *oryzae* (*Xoo*). BLS routinely reduces yield by 10–20% and causes yield losses up to 40%^3, 4^. Theoretically, the most effective way to control BLS is to grow resistant rice varieties; however, with the exception of *Rxo1* from maize^5^, only *Xo1* was reported early in 2016 as a source of resistance to African *Xoc* strain^6^. Very little novel host resistance (*R*) genes for BLS have been reported.

*Xoc* and *Xoo* show over 87% similarity when compared by DNA-DNA hybridization^2^. Despite their relatedness, the two pathovars show distinctly different infection strategies; *Xoc* colonizes the apoplast of mesophyll parenchyma cells, whereas *Xoo* invades the xylem systemically^7^. The genome sequences of *Xoc* and *Xoo* strains revealed the conservation of many pathogenicity and virulence genes between these two pathogens, such as two *Xoc* strains BLS256 (CP003057) and RS105 (CP011961) and three *Xoo* strains KACC10331 (AE013598), MAFF311018 (AP008229), and PXO99^A^ (CP000967). Both pathovars contain similar genes for exopolysaccharide (EPS) synthesis, the regulation of pathogenicity factors (*rpf*), two-component signal transduction systems, the type III secretion system (T3SS), AvrBs3/PthA family proteins that function as transcription activator-like effectors (TALEs), and other T3S effectors (T3SEs) that are translocated into host cells^8^. Genomic studies have proven helpful in understanding the tissue- and host-specificity differences between these two pathovars^9^.

The T3SEs in *X*. *oryzae* have been classified into two groups: TALEs and Non-TAL effectors (NTALEs, also nominated as Xops (*Xanthomonas* outer protein))^10^. Many NTALEs share similar features including co-regulated expression with *hrp* genes^11^, conserved N-terminal aminoacid motifs^12, 13^, and a conserved plant-inducible promoter that is bound by HrpX^14, 15^. Novel NTALEs presumably contribute to host specificity and inhibit pathogen-triggered immunity^16^.

The first reported TALE was AvrBs3, which was discovered in *X*. *campestris* pv. *vesicatoria* and triggered HR in pepper cultivars containing an *R* gene *Bs3* ^17, 18^. AvrBs3 was shown to function as a transcription factor and bound to the promoter region of *Bs3* ^19, 20^. All TALEs in *X*. *oryzae* are members of the *avrBs3*/*pthA* family^17, 21–26^, which share several features including a central repeat region containing nearly identical repeats of 34–35 amino acids. The number of repeats varies in and across strains and contributes to host specificity through repeat variable diresidues (RVDs) that recognize unique DNA codes in plants^20, 27–31^. AvrXa7, AvrXa10, and AvrXa27 are typical TALEs that elicit BLB resistance in hosts that contain the cognate *R* genes, *Xa7*, *Xa10* and *Xa27*, respectively^22, 24, 32–35^. However, interacting TALEs and cognate *R* genes have not been identified in the *Xoc*-rice pathosystem. Intriguingly, when *avrXa7* and *avrXa10* of *Xoo* strain PXO86 were transferred into *Xoc* BLS303 and BLS256, a compatible, water-soaked response was observed in rice cultivars containing the *R* genes *Xa7* and *Xa10*, respectively^36^, suggesting that *Xoc* might encode unidentified effectors that interfere with effector-triggered immunity (ETI) which might help explain the lack of effective *R* genes for resistance to BLS^36^.

In this study, we isolated *Xoc* YNB0-17, a hypovirulent strain containing nine putative *tal* genes, which is considerably smaller than the 24 *tal* genes identified in *Xoc* RS105. The heterologous expression of *avrXa7* in *Xoc* YNB0-17 but not in *Xoc* RS105, triggered ETI in rice plants containing *Xa7*. This led us to investigate whether RS105 contains unidentified effectors that enable the pathogen to suppress *avr*-*R* mediated immunity and cause disease in rice. The transfer of individual *tal* genes from *Xoc* RS105 into YNB0-17 led to the identification of *tal7*, which suppressed *avrXa7-Xa7* mediated defense. Furthermore, gel shift assays and microscale thermophoresis (MST) indicated that Tal7 bound to *Os09g29100* and *Os12g42970* promoters in rice. These two genes encode predicted Cyclin-D4-1 and GATA zinc finger family protein, respectively. Functional assays confirmed that activation of *cyclin-D4-1* could suppress AvrXa7-Xa7 mediated defense in rice.

## Results

### Heterologous expression of *Xoo tal* genes in *Xoc* YNB0-17 triggers ETI in rice

**Figure 1:**
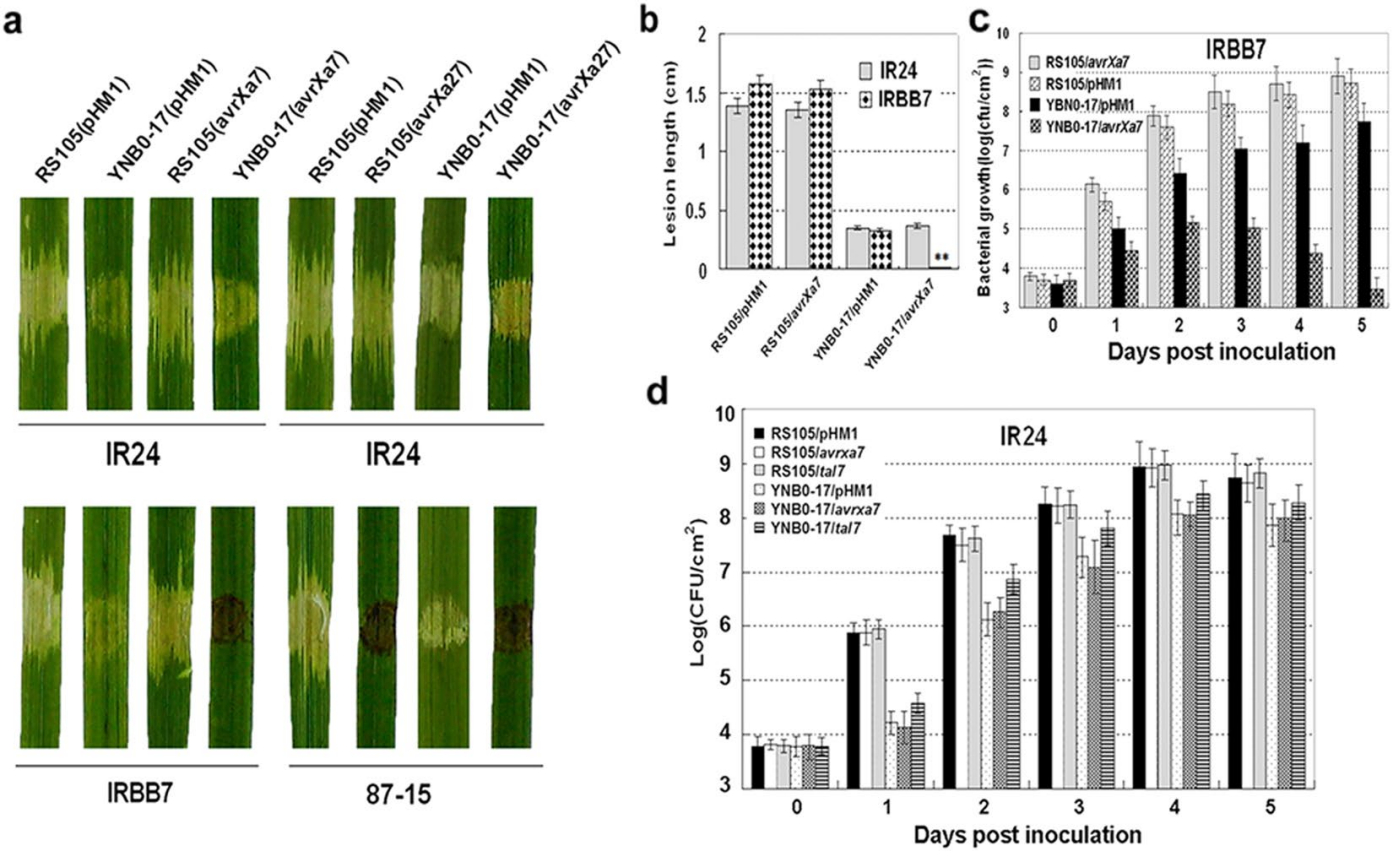
Heterologous expression of *avrXa7* and *avrXa27* in *X*. *oryzae* pv. *oryzicola* (*Xoc*) strains YNB0-17 and RS105 and inoculation to rice cv. IR24 (susceptible) and IRBB7 (contains *R* gene *Xa7*) and 87-15 (contains R gene *Xa27*). (**a**) YNB0-17 and RS105 containing *avrXa7* and *avrXa27* were infiltrated into seedlings of IRBB7 (*Xa7*) and 87-15 (*Xa27*) with needleless syringes. The susceptible rice cultivar IR24 and strains containing the empty vector pHM1 were used as controls. Leaves were scored for water-soaked symptoms or HRs within the infiltrated area 2 dpi and were designated as susceptible or resistant (showing an HR). Leaves were photographed 3 dpi. (**b**) Strains YNB0-17 and RS105 carrying *avrXa7* were inoculated to adult rice plants of IRBB7 (*Xa7*) and susceptible line IR24. Lesion lengths were recorded 14 dpi. The vertical columns and intersecting bars represent the mean lesion length and SD from five replicate plants. The asterisks in each horizontal data column indicate significant differences at *P* = 0.01 using the Student’s *t* test. (**c**) Bacterial growth in rice IRBB7 inoculated with *Xoc* YNB0-17 and RS105 containing *avrXa7* or the empty vector control (pHM1). (**d**) Bacterial growth of *Xoc* YNB0-17 and RS105 containing *avrXa7*, *tal7* or pHM1 in the BLS susceptible rice line IR24. Leaf discs (0.8 cm diameter) were excised from the inoculated areas, homogenized in sterile water, diluted and plated on to NA. Data points represent the mean ± SD from three replicates. All experiments were repeated three times, and similar results were obtained.

Multiple strains of *Xoc* were isolated and evaluated for virulence on rice cv. *Nipponbarre*. *Xoc* strain YNB0-17, which was originally isolated from Yuelianggu rice (data not shown), was hypovirulent strain on *Nipponbare* as compared to the highly virulent *Xoc* strain RS105^37^. Although endogenous avirulence-*R* gene interactions have not been identified in the *Xoc*-rice pathosystem, *Xoc* is known to contain TALEs that could potentially modulate *R* or *S* genes in rice^38, 39^. To investigate whether *Xoo tal* genes could trigger *R*-gene resistance in these strains, plasmids pHZWavrXa7 and pHZWavrXa27 (Supplemental Table S1), which contained *avrXa7* and *avrXa27*, respectively, were transferred into YNB0-17 and RS105, respectively. YNB0-17(pHZWavrXa7) and YNB0-17(pHZWavrXa27) triggered an HR when infiltrated into leaves of rice cultivars IRBB7 and 87-15, which carry resistance genes *Xa7* and *Xa27*, respectively (Fig. 1a). When the YNB0-17 derivatives were inoculated to rice cultivar IR24, typical water-soaked symptoms resulted, which is the predicted response since IR24 lacks the corresponding *R* genes (Fig. 1a). *Xoc* RS105(pHZWavrXa27) elicited an HR in rice cv. 87-15; however, inoculation with RS105(pHZWavrXa7) resulted in a susceptible (water-soaked) reaction in rice IRBB7 (Fig. 1a). Disease lesion lengths and growth curves of *Xoc* strains containing *avrXa7* are shown in Fig. 1b and c. BLS lesion lengths and growth of YNB0-17(pHZWavrXa7) in IRBB7 rice was significantly reduced in comparison to YNB0-17 containing the empty vector pHM1 (Fig. 1b and c). Remarkably, BLS lesion size and bacterial growth of RS105(pHZWavrXa7) was not significantly different from RS105(pHM1) (Fig. 1b and c). Collectively, the results shown in Fig. 1 indicated that the expression of *avrXa7* and *avrXa27* in YNB0-17 triggered an HR and decreased bacterial growth (*avrXa7*) and BLS lesion length. However, the expression of *avrXa7* in RS105 did not result in the HR and did not decrease bacterial growth *in planta*. These data suggest that like BLS256 and BLS303 strains^36^, RS105 has inhibitor(s) that may suppress *avrXa7-Xa7* ETI, and the inhibitor(s) is (are) either absent or nonfunctional in YNB0-17.

### *X*. *oryzae* pv. *oryzicola* Tal7 suppresses AvrXa7-Xa7 mediated immunity

**Figure 2:**
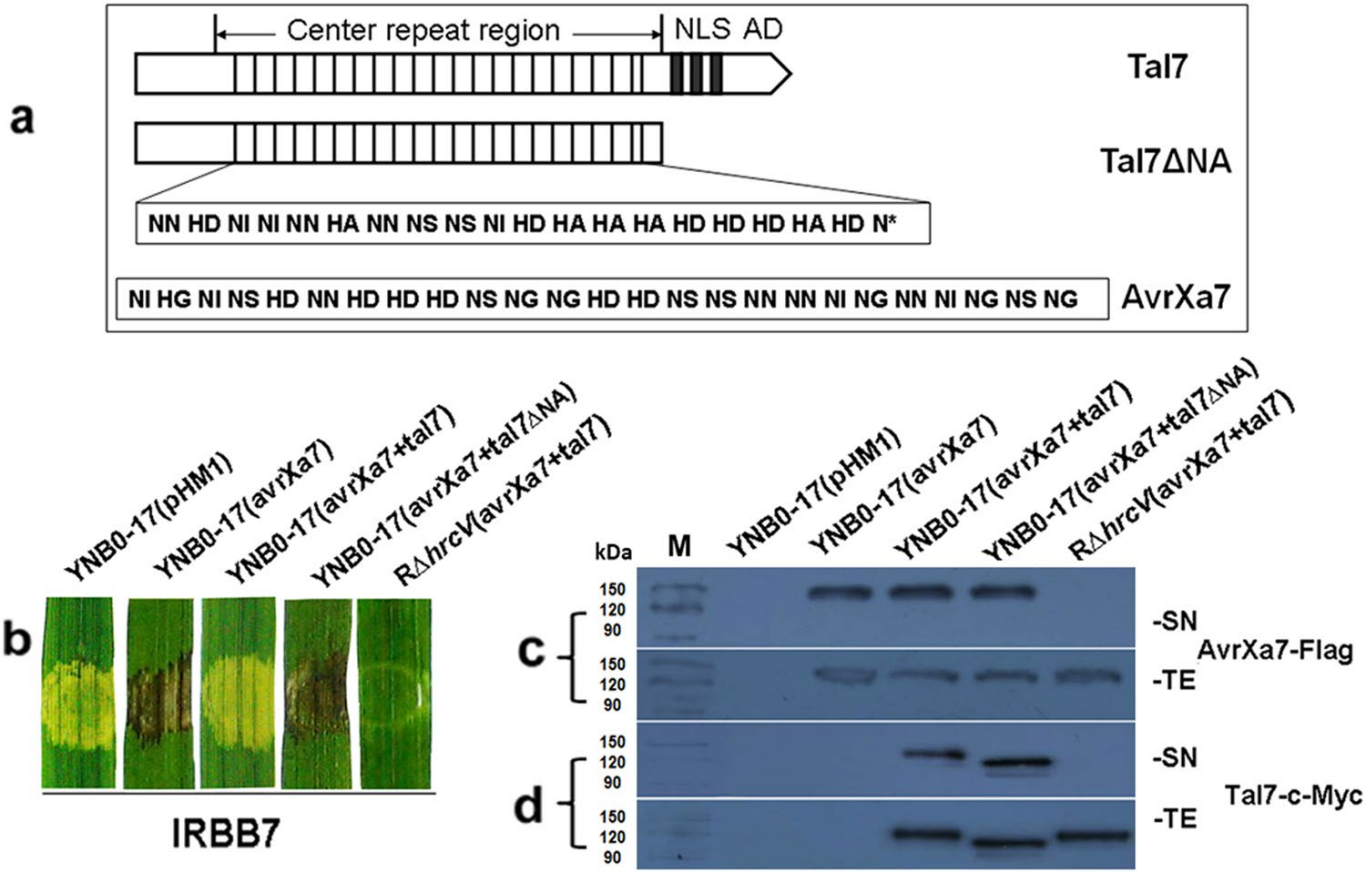
Tal7 suppresses *avrXa7*-*Xa7* mediated defense. (**a**) Diagram showing the RVDs of Tal7 and AvrXa7 and schematic construction of Tal7∆NA. (**b**) Tal7 suppressesthe *avrXa7*-*Xa7* interaction in rice. Strain YNB0-17 harboring pHM1, *avrXa7*, *avrXa7* + *tal7*, and *avrXa7* + *tal7*∆NA were infiltrated into rice seedlings of IRBB7(*Xa7*) with needleless syringes. The T3SS-deficient strain of RS105, R∆*hrcV* (Supplemental Table S1), containing *avrXa7* + *tal7*, was used as a control. Leaves were scored for water-soaked symptoms or the HR at 3 dpi and were designated as susceptible or resistant, respectively. Leaves were photographed 3 dpi. (**c**) Secretion of AvrXa7-Flag and (**d**) Tal7-c-Myc by strain YNB0-17 harboring the constructs mentioned in panel b. Bacterial cells containing p707 or p707ΔNA (Supplemental Table S1) were induced in XOM3 medium as described in Methods. Bacterial supernatants (SN) and total extracts (TE) were analyzed by SDS-PAGE, transferred to polyvinylidene difluoride membranes, and used for immunoblotting with anti-FLAG (**c**) or anti-C-Myc (**d**) as the primary antibody. The experiments were repeated three times, and similar results were obtained each time.

Comparison of the Southern blot profiles of *Xoc* RS105 and YNB0-17 indicated that the main difference between the strains was the larger number of *tal* genes in RS105 (Supplemental Fig. 1). To identify whether RS105 contains a *tal* gene that suppresses AvrXa7-Xa7 defense, we isolated individual *tal* genes from RS105 based on Southern blot analysis (Supplemental Fig. 1); the isolated fragments containing putative *tal* genes were then cloned into pUFR034 containing *avrXa7* (Supplemental Fig. 2). Remarkably, when *tal7* was co-expressed with *avrXa7* in strain YNB0-17, infiltrated tissue showed water-soaked symptoms indicative of a susceptible interaction (Fig. 2b). These results suggest that *tal7* suppresses *avrXa7-Xa7* resistance. The nuclear localization signals (NLS) and acidic transcriptional activation domains (AD) that are located at the C-terminus of TALEs and enable these proteins to function as transcription activators in plant nuclei^22, 25^. Thus, we speculated that a truncation at the C-terminus of Tal7 would impair its ability to suppress *avrXa7*-*Xa7* immunity in rice. To confirm this, we generated truncated *tal7∆*NA (Fig. 2a), which lacks the NLS and AD domains (p707*∆*NA, Supplemental Table S1). The HR was not suppressed when rice IRBB7 was infiltrated with *Xoc* YNB0-17 containing *tal7∆*NA (Fig. 2b), indicating that suppression of *avrXa7-Xa7* defense by Tal7 requires the NLS and AD domains. Immunoblotting experiments indicated that AvrXa7 was present in the supernatant (SN) and total extracts (TE) of YNB0-17 containing *avrXa7*, *avrXa7* + *tal7*, and *avrXa7* + *tal7*∆NA (Fig. 2c). Furthermore, the SN and TEs of YNB0-17 containing *avrXa7* + *tal7*, and *avrXa7* + *tal7∆*NA also contained Tal7 (Fig. 2d). The absence of AvrXa7 and Tal7 in the supernatant of the T3SS mutant, R*∆hrcV* (Fig. 2c and d), indicates that both effectors are delivered into plant cells via T3SS.

### Tal7 targets rice genes *Os09g29100* and *Os12g42970*

**Figure 3:**
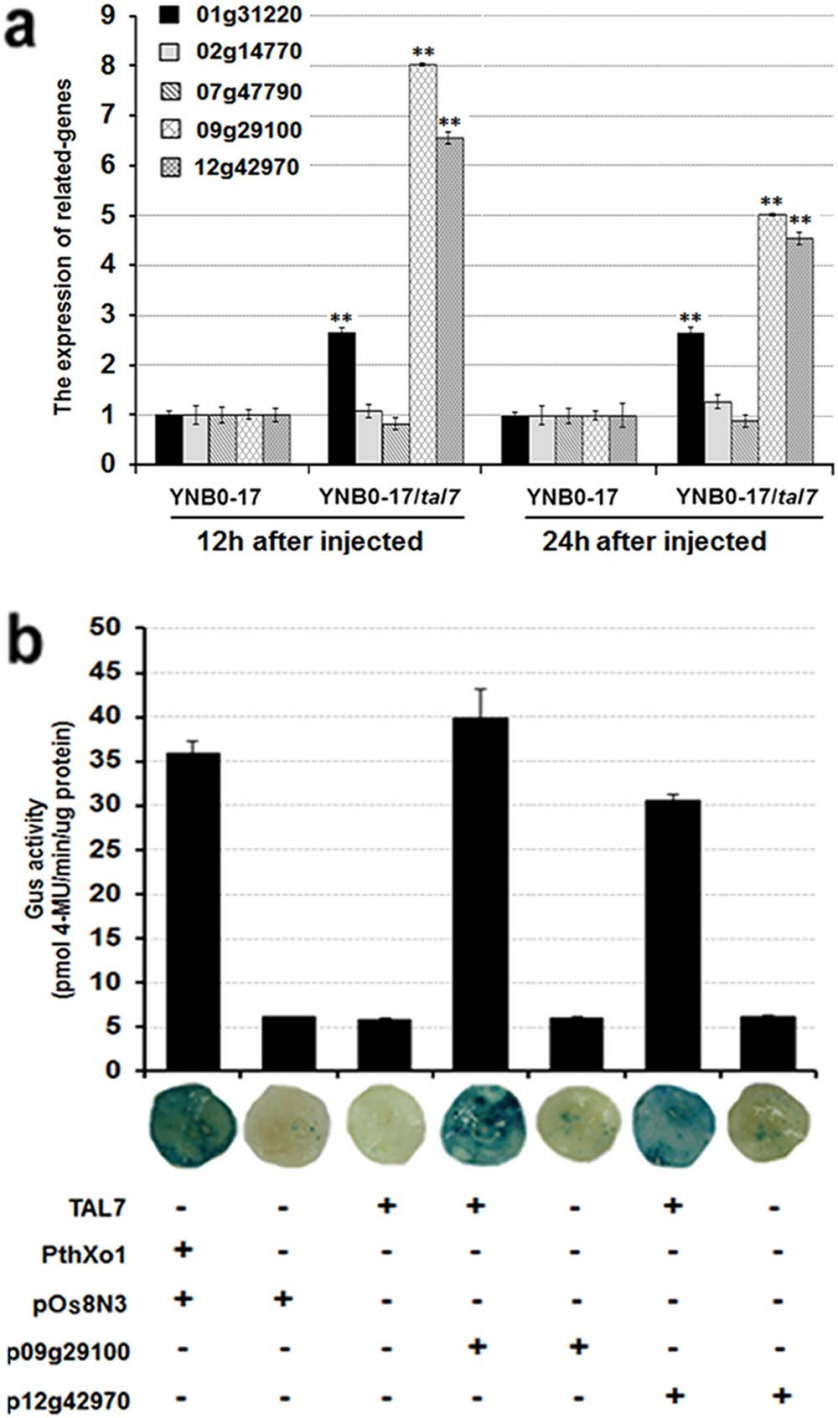
*Xoc* Tal7 targets rice genes *Os09g29100* and *Os12g42970*. (**a**) The expression of candidate targets of Tal7. *Xoc* strains YNB0-17, and YNB0-17(*tal7*) were infiltrated into IR24 rice, and the expression of five rice genes (*Os01g31220*, *Os02g14770*, *Os07g47790*, *Os09g29100*, and *Os12g42970*) was measured 12 and 24 hpi by real time qRT-PCR. The expression levels of *Actin* and *18S rRNA* used as internal standards. The asterisks in each horizontal data column indicate significant differences at *P* = 0.01 using the Student’s *t* test. Data are the mean ± SD of triplicate measurements from a representative experiment; and similar results were obtained in two other independent experiments. (**b**) Transcriptional activation of rice genes *Os09g29100* and *Os12g42970* by TALEs of *Xoc* Tal7. The TALE PthXo1 from *Xoo* and its target rice gene, *Os8N3* (pOs8N3), were used as a positive control. Reporter fusions containing the rice promoters fused to GUS were codelivered via *A*. *tumefaciens* into *N*. *benthamiana* with (+) and without (−) constructs encoding TAL7 and PthXo1. pOs8N3, p09G29100 and p12g42970 represent the *Os8N3*, *09g29100* and *12g42970* promoters fused to GUS (see Methods). For quantitative assays, two leaf discs (0.9 cm diameter) were sampled 2 dpi, and GUS activity was determined using 4-methyl-umbelliferyl-β-D-glucuronide (MUG). 4-MU is 4-methyl-umbelliferone. Error bars indicate standard deviations (n = 3 samples from different plants). For qualitative assays, GUS activity in excised leaf discs was determined 3 dpi with X-Gluc (5-bromo-4-chloro-3-indolyl-b-D-glucuronide). A blue color indicatesa positive reaction. All experiments were performed twice with similar results.

Cernadas *et al*.^38^ demonstrated that up-regulation of *Os01g31220*, *Os02g14770*, *Os07g47790*, *Os09g29100* and *Os12g42970* genes depends on Tal7 of BLS256 (in that paper *tal7* was nominated as *tal6*). Two of these genes, *Os09g29100* and *Os12g42970*, which encode predicted Cyclin-D4-1 and GATA zinc finger family protein, respectively, are among the most highly-induced genes when YNB0-17(*tal7*) were infiltrated into IR24 rice (Fig. 3a). *Os09g29100* expression was 8-fold (12 hpi) and 5-fold (24 hpi) higher than the control (YNB0-17) after *Xoc* YNB0-17(*tal7*) was infiltrated to IR24 rice; whereas *Os12g42970* expression was 6.5- and 4.5-fold higher at 12 hpi and 24 hpi, respectively (Fig. 3a). These data suggest that *Os09g29100* and *Os12g42970* are possibly the Tal7 targets and others are not.

To validate that Tal7 activates the expression of selected rice genes, 500-bp fragments containing the promoter regions of *Os09g29100* and *Os12g42970* were fused to the promoterless *gusA* gene and cloned in pCAMBIA1381 (Supplemental Table S1, p09g29100 and p12g42970). *Agrobacterium* strains containing GUS reporter constructs and *tal* genes were co-transformed into *N*. *benthamiana* leaves (see Methods). The *Os8N3* promoter fused to *gusA* (Supplemental Table S1, pOs8N3) and a *tal* gene *pthXo1* were included as controls (Fig. 3b). Tal7 triggered *Os09g29100*- and *Os12g42970*-mediated GUS activity. Quantitative assays indicated that GUS activation was higher for the *Os09g29100* promoter (Fig. 3b), potentially due to weaker effector binding or/and reduced activation of the GATA zinc finger family protein gene promoter.

### Tal7 binds EBEs of rice genes *Os09g29100* and *Os12g42970*

As described above, Tal7 induced expression of *Os09g29100* and *Os12g42970*. Thus, it was tempting to speculate that Tal7 targets the promoter regions of the two genes in rice. We then examined the *Os09g29100* and *Os12g42970* promoters to validate the predicted Tal7 binding site (EBE_*tal7*_). By examining the RVDs of Tal7 in the context of the TALE recognition code, which governs the interaction between TALE RVDs and their target nucleotides^20, 27^, we could visually corroborate the predicted EBEs for Tal7 in the *Os09g29100* and *Os12g42970* promoter regions using the TALE-NT program (http://tale-nt.cac.cornell.edu/, Supplemental Fig. 3a,b). The two promoter regions each contained a similar sequence, and the predicted binding sequence was identified with most of the identical nucleotides in the 5′ region (Supplemental Fig. 3a,b).

**Figure 4:**
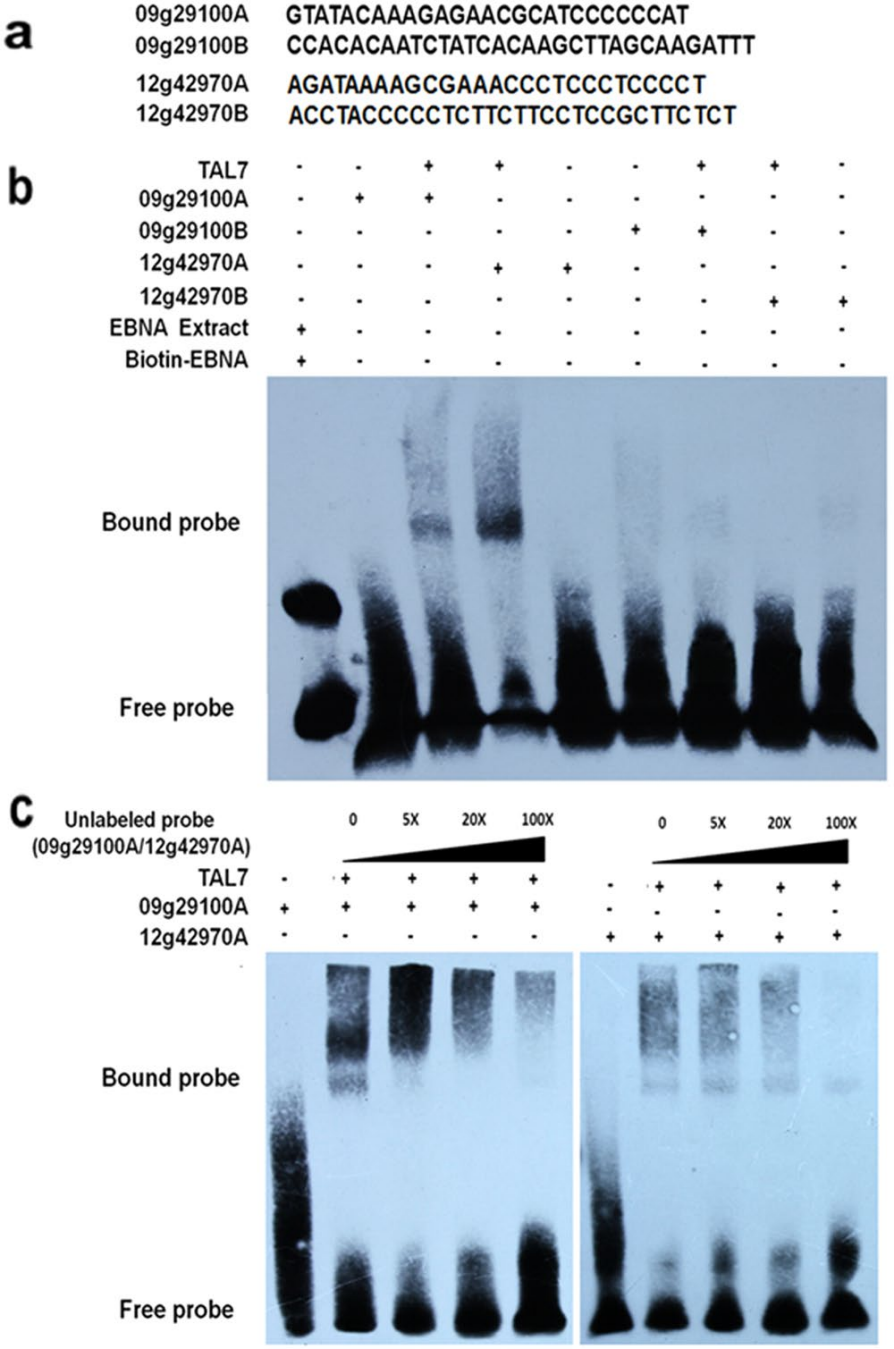
*Xoc* Tal7 binds the EBEs of the *Os09g29100* and *Os12g42970* promoter regions. Electromobility shift assays (EMSA) were performed using 20 fM biotin-labeled DNA fragments derived from the two promoter regions as probes. Unlabeled probes were used as competitor DNA. The presence of DNA or protein is indicated by (+), and absence by (−). (**a**) The DNA sequences of the four probes for EMSA, 09g29100A, 09g29100B, 12g42970A and 12g42970B, are shown at the top of the panel. (**b**) EMSA results for the probes from *Os09g29100* and *Os12g42970*. EBNA extract protein and biotin-EBNA coming from EMSA Kit (Thermo, USA) used as positive contral. (**c**) Competition of the biotinylated probes with unlabeled incubated with His-Tal7. Unlabeled probe 09g29100A and 12g42970A were incubated at increasingly higher concentrations; e.g. 5, 20, and 100 X times more than the 20 fM of biotinylated 09g29100A and 12g42970A, respectively. The experiments were repeated two or more times with similar results.

Potential binding of Tal7 to the candidate EBE_*tal7*_ sequences was investigated by EMSA using His-Tal7 fusion proteins and biotin-labeled *Os09g29100* and *Os12g42970* promoter fragments (Supplemental Fig. 3a,b). These assays showed that Tal7 bound to fragments 09g29100A and 12g42970A, but not 09g29100B and 12g42970B (Fig. 4a). The specificity of binding was further confirmed by competition assays with labeled and unlabeled (0, 5X, 20X, 100X) 09g29100A and 12g42970A fragments (Fig. 4b). These results indicate that Tal7 bind *Os09g29100* and *Os12g42970* promoters at the EBE_*tal7*_ location (fragments 09g29100A and 12g42970A, Fig. 4a).

MST (Supplemental Methods S3) supported the EMSA data and indicated that Tal7 interacted with the *Os09g29100* and *Os12g42970* promoter regions (Supplemental Fig. 4). The K_d_ values for the Tal7-09g29100 and Tal7-12g42970 interactions were 2.26 and 2.926 µM (Supplemental Fig. 4). AvrXa7 showed a binding affinity for *Os11N3*, which was used as a positive control (Supplemental Fig. 4) and Tal7 binding affinity for *Os11N3* was used as a negative control (Supplemental Fig. 4).

### Activation of *Os09g29100* results in the suppression of AvrXa7-Xa7 ETI

*Xoc* strain RS105, containing *tal7* (genomic copy) and *avrXa7* (*in trans*), failed to elicit an HR in IRBB7 rice containing the *R* gene *Xa7* (Fig. 1a). This led us to speculate that Tal7 was responsible for suppressing AvrXa7-Xa7 ETI. The EMSA and MST experiments both indicated that Tal7 bind the EBEs in the promoters of *Os09g29100* and *Os12g42970* (Fig. 4 and Supplemental Fig. 4); consequently, experiments were designed to determine if the Tal7-EBE_*tal7*_ interaction might induce target genes expression and then suppress AvrXa7-Xa7 ETI.

**Figure 5:**
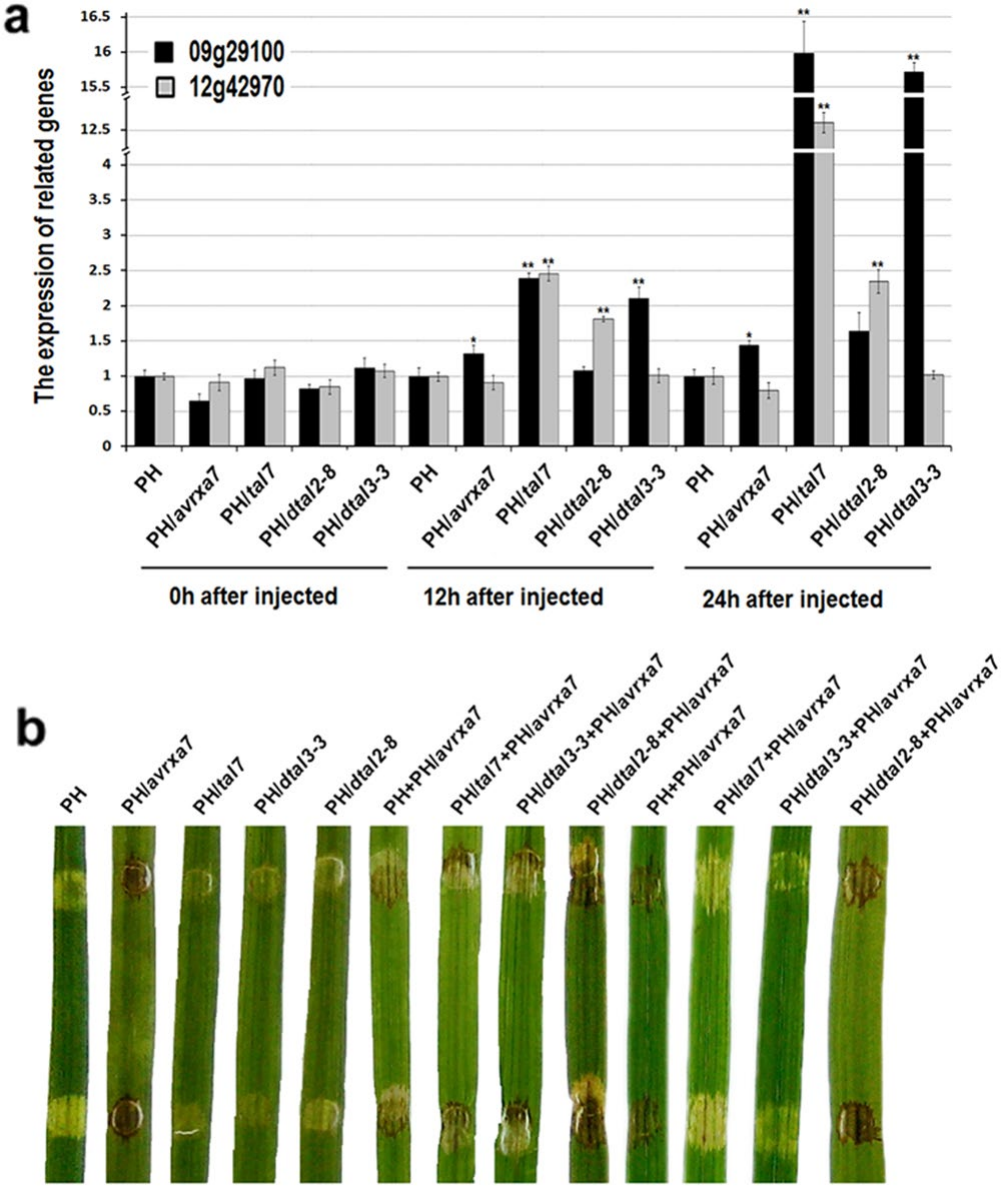
*Os09g29100* is a major susceptibility gene in rice that can mask or suppress AvrXa7-Xa7 ETI. Strain PH, a tal-free strain of *Xoo* (Supplemental Table S1) was used in these experiments. (**a**) Expression of rice genes *Os09g29100* and *Os12g42970 in planta* by real-time qRT-PCR. *Xoo* strain PH, PH/avrXa7, PH/tal7, PH/dtal3-3 and PH/dtal2-8 were infiltrated into IR24 rice leaves, and expression of *Os09g29100* and *Os12g42970* was evaluated 0, 12 and 24 hpi. Data are the mean ± SD of triplicate measurements from a representative experiment, and similar results were obtained in two other independent experiments. The asterisks in each column indicate significant differences at *P* = 0.01 by *t* test. (**b**) Role of Tal7 and two dTALEs in suppressing *avrXa7*-*Xa7* ETI. *Xoo* strain PH and PH harboring *avrXa7*, *tal7*, dtal3-3 or dtal2-8 were infiltrated into rice seedlings of IRBB7 (*Xa7*) with needleless syringes. Images show inoculation with a single strain (five panels on left), one strain followed 3 h later with another strain (four middle panels), and two co-infiltrated strains (four panels at right). Leaves were scored for water-soaked symptoms or the HR within the infiltrated area 3 dpi and were designated as susceptible or resistant (showing an HR). Leaves were photographed 3 dpi.

To evaluate which rice gene is the biologically relevant, we engineered two designer TAL effectors (dTALEs), dtal2-8 and dtal3-3 (Supplemental Table S1), to specifically bind *Os09g29100* and *Os12g42970* promoter regions outside EBE_*tal7*_ loci (Supplemental Fig. 3a,b). To investigate whether the two dTALEs can also suppress AvrXa7-Xa7 ETI in IRBB7 rice, the constructs for co-expression of *dtal2-8* or *dtal3-3* with *avrXa7* were transferred into YNB0-17 and infiltrated rice tissue showed only *dtal2-8* suppressed Xa7 defense (data not shown). To exclude possible interference of other TALEs in AvrXa7-Xa7 defense, an *Xoo tal*-free strain PH^40^ of PXO99^A^ were transferred with the plasmids pHZWavrXa7, pHZWtal7, pHZWdtal2-8 and pHZWdtal3-3 containing *avrXa7*, *tal7*, *dtal2-8* and *dtal3-3*, respectively and then were inoculated in rice with needleless syringes. First, *Os09g29100* and *Os12g42970* gene expression levels in IR24 rice were evaluated after PH, PH(*avrXa7*), PH(*tal7*), PH(*dtal2-8*) and PH(*dtal3-3*) were infiltrated at 0, 12, and 24 h. Real-time qPCR assays indicated that *dtal2-8* and *dtal3*-3 specifically and significantly activated the expression of *Os09g29100* and *Os12g42970* genes, respectively, beginning 12 hpi (Fig. 5a). Functional assays indicated that only *dtal3-3* suppressed AvrXa7-Xa7 ETI in a manner analogous to *tal7* (Fig. 5b). Suppression of AvrXa7-Xa7 ETI was only observed when *dtal3-3* and *avrXa7* were co-expressed (e.g. PH/*dtal3-3* + PH/*avrXa7*; Fig. 5b). Collectively, these data indicate that only *Os09g29100*, which encodes Cyclin-D4-1, is the relevant Tal7 target, and the activation of *cyclin-D4-1* may suppress AvrXa7-Xa7 defense in IRBB7 rice.

### TALEN-mediated disruption of EBE_*tal7*_ in *cyclin-D4-1*

**Figure 6:**
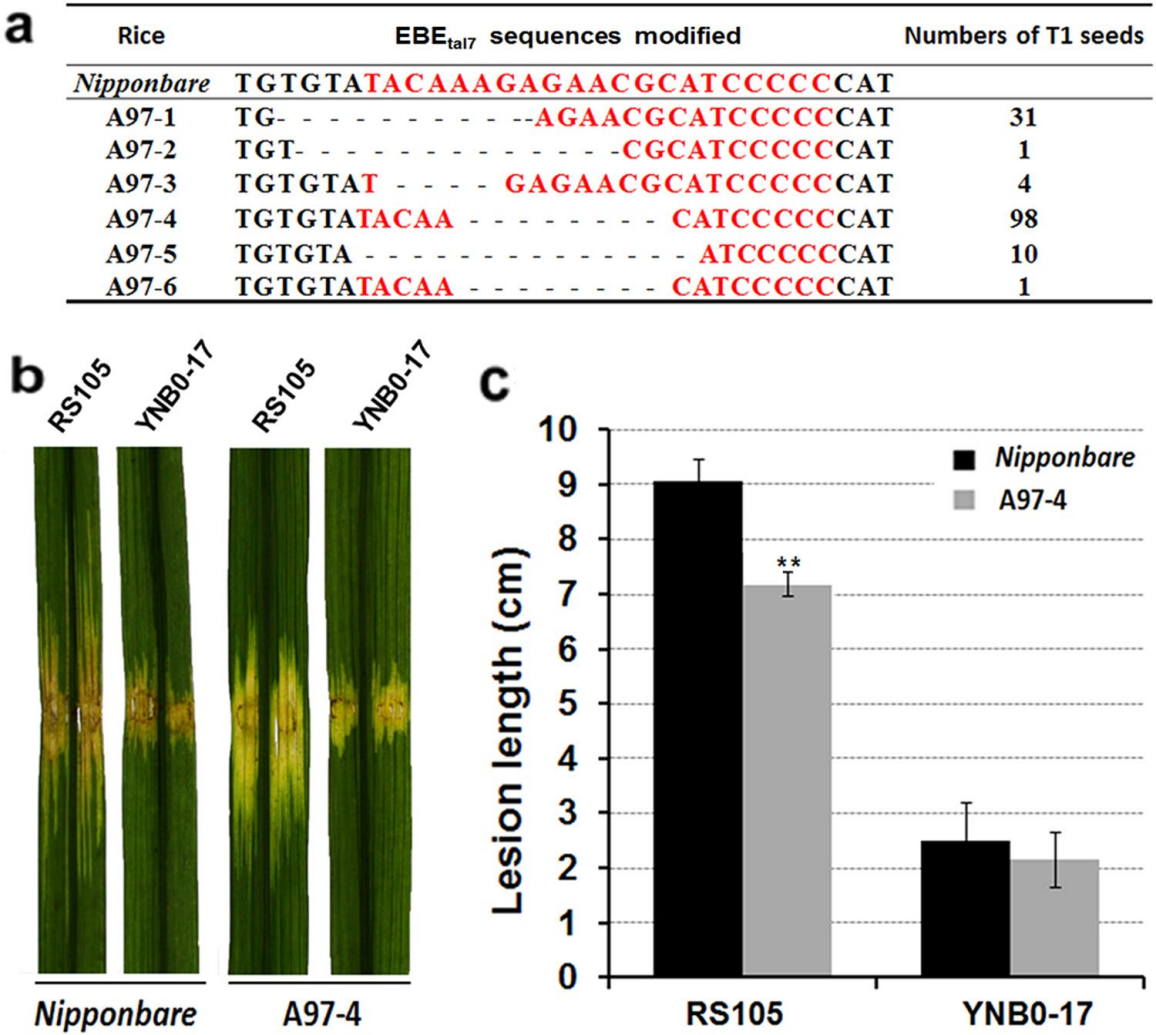
TALEN-modified editing of EBE_*tal7*_ in rice gene Os*09g29100*. (**a**) Colored letters indicate the nucleotide preference of Tal7 according to the TALE code. Letter height represents the preferences relative to other nucleotides for the RVD (generated with TALGetter: http://galaxy.informatik.uni-halle.de/root?tool_id=TALgetter). The RVDs of Tal7 DNA-binding domain and nucleotide sequence of the EBE_*tal7*_ region in *Os09g29100* are shown. The table shows the EBE_*tal7*_ sequences in *Nipponbare* and six TALEN-edited lines (A97-1, A97-2, A97-3, A97-4, A97-5, and A97-6). The EBE_*tal7*_ sequence is shown in red font; the dashed lines (−) indicate deletions obtained from TALEN editing. The number of T1 seeds obtained from each line is indicated. (**b**) Inoculation of *Xoc* RS105 (hypervirulent) and YNB0-17 (hypovirulent) to rice cv. *Nipponbare* and transgenic line A97-4 containing the TALEN-modified EBE_*tal7*_ in *Os09g29100*. Strains YNB0-17 and RS105 (OD_600_ = 0.5) were inoculated to adult rice plants with needleless syringes and photographed 14 dpi. (**c**) Disease lesion lengths on cv. *Nipponbare* and rice transgenic line A97-4 at 14 dpi. Column height represents mean lesion length and vertical bars show the ± SD from five replicate plants. All experiments were repeated three times, and similar results were obtained.

A pair of TALENs was used to induce mutations in EBE_*tal7*_ of rice gene *Os09g29100*. The TALEN pair was designed to recognize sequences on both sides of EB*E*_*tal7*_ (Supplemental Fig. 5). After failure in obtaining transgenic plants from IRBB7, forty transgenic plants (T_0_) from the donor rice *Nipponbare* were obtained after transformation with pCAMBIA1301-EBE_*tal7*_ and grown to maturity. Seeds (T_1_, n = 145) were cultivated and leaves were harvested for analysis of potential mutations in EBE_*tal7*_. Digestion with TE7I to detect TALEN-mediated mutations^41^ and sequence analysis confirmed the existence of six TALEN-edited lines, which were designated A97-1, A97-2, A97-3, A97-4, and A97-5, and A97-6 (Fig. 6a). There were no differences in resistance phenotypes of the six T_1_ transgenic lines (data not shown). Consequently, line A97-4 was used for further study since more seed were obtained from this line.

*Xoc* RS105 and YNB0-17 were inoculated to rice cv. *Nipponbare* and transgenic line A97-4 containing the TALEN-modified EBE_*tal7*_ in *Os09g29100*. The hypervirulent RS105 showed reduced symptom severity on transgenic line A97-4 as compared to *Nipponbare*, whereas the hypovirulent YNB0-17 did not (Fig. 6b and c), strongly suggesting that *Os09g29100* is the target of Tal7 contributing to the BLS development.

## Discussion

*Xoc* and *Xoo* show a high level of DNA/DNA similarity, but have very different infection strategies^3, 42^. Although both *Xoc* and *Xoo* contain TALEs, effector/*R* gene interactions have not been described in *Xoc*-rice pathosystem. In the present study, we focused on the hypervirulent *Xoc* strain RS105 and hypovirulent YNB0-17. The latter strain was isolated from rice cv. *Yuelianggu* in the remote mountainous regions of the Yunnan province and could be a progenitor of more highly-evolved *Xoc* strains. The *Xoo avrXa7* and *avrXa27* were transferred into *Xoc* YNB0-17 and RS105; as expected, YNB0-17 containing *avrXa7* and *avrXa27* elicited the HR in rice containing *R* genes *Xa7* and *Xa27*, respectively (Fig. 1a). *Xoc* RS105 expressing *avrXa27* also elicited an HR in *Xa27* rice; however, a compatible, virulent phenotype was observed when RS105/*avrXa7* was inoculated to *Xa7* rice (Fig. 1a). These results are reminiscent of those reported previously where the expression of *avrXa7* and *avrXa10* in *Xoc* BLS256 and BLS303 failed to elicit *Xa7* mediated defense^36^.

We speculated that unidentified TALEs or NTALEs exist in *Xoc* RS105 that enabled the strain to suppress ETI and cause disease in rice. Since *Xoc* YNB0-17 lacks *avrRxo1* and *xopO*, which are present in RS105 (Supplemental Fig. 6a,b), we investigated whether these two effectors suppress ETI in YNB0-17 when co-expressed with *avrXa7*. The co-expression of *avrRxo1* or *xopO* in YNB0-17/avrXa7 did not inhibit *avrXa7-Xa7* mediated ETI (Supplemental Fig. 6c,d), which prompted us to undertake a more global search for unidentified TALE(s) in RS105. Individual *tal* genes were cloned from *Xoc* RS105 based on Southern blot experiments (Supplemental Fig. 1) and transferred into YNB0-17 individually. This approach led to the discovery of Tal7, which suppressed AvrXa7-Xa7 mediated immunity in YNB0-17 expressing *avrXa7* (Fig. 2b). This leads us to postulate that more virulent *Xoc* strains like RS105 have acquired a larger, more diverse repertoire of *tal* genes so they can bypass, circumvent, or suppress ETI.

Cernadas *et al*.^38^ reported five rice genes, *Os01g31220*, *Os02* *g14770*, *Os07g47790*, *Os09g29100* and *Os12* *g42970*, as predicted targets of Tal7. In our study, the expression of *Os09g29100* and *Os12g42970* were significantly up-regulated than other three when IR24 rice was infiltrated with YNB0-17/*tal7* (Fig. 3a), suggesting that the two genes are Tal7 target genes in rice.

We then used the TALE-NT program^43^ to identify potential EBEs recognized by Tal7 (EBE_*tal7*_) in the promoters of *Os09g29100* and *Os12g42970*. Four EBE_*tal7*_ sequences were found in the promoters of these two genes (Supplemental Fig. 3a,b) and were designated 09g29100a, 09g29100b, 12g42970a, and 12g42970b (Fig. 4a). Interestingly, Cernadas *et al*.^38^ also predicted two candidate EBEs for Tal7 in the promoter of *Os12g42970* but not *Os09g29100*. In the currently study, EMSA indicated that Tal7 bound 09g29100a and12g42970a, but not 09g29100b or 12g42970b (Fig. 4a).

Although the results indicated that Tal7 bound the promoter regions of both 09g29100A and 12g42970A, it wasn’t clear which interaction was significant in the suppression of AvrXa7-Xa7 ETI. This was investigated by engineering *dtal3-3* and *dtal2-8* for specific activation of *Os09g29100* and *Os12g42970*, respectively (Supplemental Fig. 3). The two dTALEs were introduced into *Xanthomonas* strains YNB0-17 and PH, and evaluated for suppression of AvrXa7-Xa7 ETI in IRBB7 rice. Functional assays indicated that only dtal3-3 suppressed AvrXa7-Xa7 ETI in a manner analogous to *tal7* (Fig. 5b). More importantly, these results indicate that *Os09g29100* is the biologically relevant target of Tal7 with respect to suppression of ETI; this is evident because dtal3-3 (but not dtal2-8) targets *Os09g29100* promoter. This study further illustrates the discriminatory ability of dTALEs to target specific genes and assess biological relevance, which has been validated in other *Xanthomonas*-plant interactions^38, 44, 45^.

Numerous effectors are produced by microbial pathogens and many are known to promote disease by suppressing plant defense^46^. In the present study, we demonstrated that Tal7 from *Xoc* suppressed AvrXa7-Xa7 mediated defense. Tal7 exhibits the modular structure typical of TALEs (Fig. 2a) and contains highly conserved C-terminal domains with NLS and AD features; both the NLS and AD domains of TALEs are known to be required for activation of plant gene expression^47^. Correspondingly, a truncated form of Tal7 lacking the NLS and AD domains (Tal7ΔNA) failed to suppress *avrXa7*-*Xa7* mediated immunity in *Xa7* rice; instead, an HR (defense) was observed in *Xa7* rice (Fig. 2b), indicating that transcriptional activation of the target rice gene *Os09g29100* is required for suppression of the defense response. In this regard, *Os09g29100*, which encodes a Cyclin-D4-1 protein, may promote BLS development in rice, since the TALEN-modified rice in *Os09g29100* promoter enhanced resistance to the hypervirulent RS105 containing *tal7* (Fig. 6). This is consistent with the previous observation that the mutation in *tal7* of BLS256 strain decreased *Xoc* virulence in rice *Nipponbare* where the *Os09g29100* gene is present^38^. We assume that the presence of Tal7 in *Xoc* strains leads to rice parenchyma cells to produce more nutrients to fit *Xoc* proliferation, since Cyclin-D4-1 is a member of the cyclin protein family that is involved in regulating cell cycle progression and putatively bind to and activate protein kinases named cyclin-dependent kinases (CDKs)^48^. The activation of CDKs in rice may alter signal induction in HR induction in rice mediated by AvrXa7-Xa7. Nevertheless, this needs first the cloning of *Xa7* from IRBB7 and the generation of mutants either in Cyclin-D4-1 or/and Xa7 in IRBB7 rice.

Several TALEs produced by the related pathogen *Xoo* are known to target rice susceptibility genes in the SWEET family variety of plant genes. SWEET susceptibility genes (e.g. *Os8N3* and *OS11N3*) are known to export sugars from the plant cell, a process that supports bacterial proliferation^30, 44, 45, 49^. It is likely that the *S* genes in rice that are targeted by *Xoc* TALEs differ from those produced by *Xoo* ^50^. Recently, Cernadas *et al*.^38^ identified Tal2g, which is an *Xoc* TALE that targets an *S* gene *OsSULTR3;6*, a predicted sulfate transporter. Interestingly, Tal2g did not suppress *avrX7-Xa7* defense in our investigation (data not shown).

Our results indicate that Tal7 activated *Os09g29100* expression, leading to suppress *avrXa7-Xa7* defence in rice (Figs 3a, 2b and 5). Multiple TALEs have been shown to bind to the promoters of *S* genes in rice; for example, overlapping EBEs have been identified in the *Os11N3* and *Os8N3* promoter regions^30, 49–51^. Our data indicate that genetic modification of the EBE_*tal7*_ binding site via TALEN editing could be deployed to reduce Tal7 binding, which could potentially reduce disease severity (Fig. 6b and c). This approach shows great promise in engineering rice cultivars with reduced susceptibility to *X*. *oryzae* ^52–54^.

Over 40 distinct BLB resistance genes have been identified from cultivated rice varieties and wild relatives, and many *R* genes have been used in breeding programs for disease control^10, 55, 56^. Interestingly, *R* genes resistant to *Xoo* infection are ineffective to *Xoc*, and several factors may explain this phenomenon. (i) *Xoc* lacks the cognate *tal* genes that interact with BLB *R* genes; for example, *Xoc* strains RS105 and YNB0-17 do not encode *avrXa7* and *avrXa27*. (ii) *Xoc* has major TALEs activating *S* gene expression that differs from *Xoo*. (iii) *Xoc* TALEs may function to suppress BLB resistance, such as Tal7 in the present study. Continued efforts to decipher *tal-*gene functions will increase our understanding of BLS and BLB in rice and will enhance our ability to control these diseases via *R* genes.

## Materials and Methods

### Bacterial strains, plasmids and growth conditions

The bacterial strains and plasmids used in this study are listed in Supplemental Table S1. *Escherichia coli* strains were cultivated in Luria-Bertani medium^57^ at 37 °C. All *X*. *oryzae* strains were grown in nutrient agar or NB broth (NA without agar) at 28 °C^58^. Antibiotics were used at the following final concentrations as required: ampicillin, 100 μg ml^−1^; rifampicin, 75 μg ml^−1^; kanamycin, 25 μg ml^−1^; spectinomycin, 50 μg ml^−1^.

### DNA manipulation

DNA isolations, restriction enzyme digestions, electroporation, PCR and Southern blots were performed according to standard procedures^59^. The primers used for PCR are listed in Supplemental Table S1. Fragments of *tal* genes were verified by sequence analysis and analyzed with Vector NTI software (http://www.invitrogen.com).

To express *avrXa7* in *X*. *oryzae* strains, primers were utilized to amplify a 280-bp conserved promoter (*tal-*pF and *tal-*pR) (Supplemental Table S2) and a 210-bp terminal region (*tal*-tF and *tal*-tR) (Supplemental Table S2) from *Xoo* PXO99^A^. These regions are identical in *tal* loci in both *Xoo* and *Xoc* (data not shown). The amplified products were digested with *Kpn* I and *Bam*H I and fused; the resulting fragment was amplified again using *tal*-pF and *tal*-tR and cloned into pMD18-T (Takara, Dalian, China). The *Bam*H I fragment containing *avrXa7*, which was obtained from pHZWavrXa7 (Supplemental Table S1), was inserted into pMD18-T containing the fused fragments. The *Kpn *I fragment from the above constructs was transferred into vector pUFR034, generating pavrXa7 (Supplemental Table S1).

To isolate *tal* genes from RS105, genomic DNA was digested with *Bam*H I and subjected to electrophoresis in 1.2% agarose gels (Supplemental Methods S1). The region containing *tal* genes was gel-purified and ligated into *Bam*H I-digested pBluescriptII SK(−) for *in situ* hybridization, digestion and sequencing. *tal* genes of interest were then transferred into pavrXa7 to replace *avrXa7* at *Bam*H I sites. To identify a *tal* from RS105 strain that may suppress *avrXa7*-*Xa7* defense, a vector was constructed using cluster 2 of *tal* genes in strain PXO99^A^ ^60^ as the basic frame (Supplemental Fig. 1). Four primers pairs (*tal-*KHKF/*tal*-KHKR, *tal*-KHHF/*tal*-KHHR, *tal-*HKHF/*tal*-HKHR and *tal*-HKKF/*tal*-HKKR; Supplemental Table S2) were synthesized to amplify the sequences from *pthXo1* and *tal2a* (Supplemental Fig. 2). Genomic DNA of PXO99^A^ was used as template, and a *Hin*d III site was created by a single nucleotide mutation (G → C) (Supplemental Fig. 2). The *Bam*H I fragment of *avrXa7* was inserted into *pthXo1* and ligated into pMD18-T, thus assembling the KH1 and KH2 fragments. Later, single *Bam*H I fragments of *tal* genes isolated from RS105 were individually introduced into the *tal2a* locus flanked by HK1 and HK2 (Supplemental Fig. 2). The new frame harboring *avrXa7* and a single *tal* candidate from RS105 strain was generated by fusion at the *Hin*d III site (Supplemental Fig. 2). The fusions were then transferred into pUFR034 at *Kpn* I sites, and the constructs were transformed into YNB0-17 for further investigation.

### Plant assays

*Nipponbare* (*Oryzae sativa* sp. *japonica*) and IR24 are BLB- and BLS-susceptible lines. IRBB7 (*Xa7*) and 87-15 (*Xa27*) are rice lines carrying different resistance genes for BLB^32, 50^. These lines were used for assaying the response to *avrXa7* and *avrXa27* to monitor the virulence and avirulence of *X*. *oryzae* strains containing different *tal* genes. Plant experiments were conducted in the greenhouse at Shanghai Jiao Tong University. Rice plants were grown under 14 h light (30 °C)/10 h dark (25 °C) conditions. Bacterial cultures were grown overnight, washed twice, and resuspended to an OD_600_ = 0.6 in sterile distilled water for inoculation to rice. Four-week old rice seedlings were inoculated by infiltrating secondary leaves with a needleless syringe^58^. Water-soaked symptoms or an HR were recorded two days post inoculation (dpi). Virulence assays on adult rice plants (two months old) were performed by leaf-needling with bacterial suspensions at OD_600_ = 0.6, and lesion lengths were measured 14 dpi. Ten leaves were used for each strain in each experiment, and independent experiments were conducted at least three times.

### Measurement of bacterial growth in rice

Bacterial suspensions (OD_600_ = 0.6) were infiltrated into secondary leaves of four-week old rice seedlings using needleless syringes. Three 0.8 cm diameter leaf discs were harvested with a cork borer from each area after infiltration. After sterilization in 20% sodium hypochlorite, 75% ethanol and sterile water, the discs were ground with a sterile mortar and pestle in 1 ml of distilled water, diluted, and plated to determine the cfu cm^−2^. Serial dilutions were spotted in triplicate on NA with appropriate antibiotics. Plates were incubated at 28 °C for 3–4 days until single colonies could be counted. The cfu cm^−2^ was estimated, and the standard deviation (SD) was calculated using colony counts from the three replicate spots of three samples taken at each time point. Experiments were repeated at least three times.

### Immunoblotting assays

Immunoblotting with Flag and c-Myc labeled antibodies was used to detect the secretion of AvrXa7-Flag and Tal7-c-Myc by *Xoc* YNB0-17. AvrXa7 was cloned in frame with C-terminal 3X FLAG-tag epitopes, and Tal7 and Tal7∆NA were cloned with C-terminal 3X c-Myc-tag epitopes. YNB0-17 bacterial cells containing the C-terminally tagged *tal* genes in constructs p707 and p707ΔNA (Supplemental Table S1) were cultured in NB to the logarithmic phase. The harvested bacteria were washed twice, and the OD_600_ was adjusted to 2.0 with sterile distilled water. The bacterial suspension (1 ml) was added to 40 ml filter-sterilized XOM3 medium^61^ and incubated at 28 °C for 6 h. The medium was centrifuged to separate total extracts (TE) and supernatant fractions (SN), and secreted proteins in the supernatant were precipitated with 12.5% trichloroacetic acid^62^. Proteins were separated on 8% SDS-PAGE gels and transferred to polyvinylidene difluoride membranes for immunoblotting using anti-FLAG or anti-C-Myc (Transgene, Beijing, China) as the primary antibody. Primary antibodies were detected using goat anti-rabbit IgG (H + L) (Transgene) and visualized with the EasySee Western Kit (Transgene).

### Real-time quantitative RT-PCR

Real-time quantitative PCR was conducted to evaluate gene expression in different culture conditions using the Applied Biosystems 7500 Real-time PCR System and SYBR Premix-Ex Taq (TaKaRa). The SV Total RNA Isolation System (Promega, USA) was used to isolate RNA from rice leaves inoculated with *X*. *oryzae*. cDNA fragments were synthesized using the Revert Aid First Strand cDNA Synthesis Kit (TaKaRa). The resulting first-strand cDNA was diluted to a final volume of 20 μl, and SYBR green-labeled PCR fragments were amplified using primers designed to amplify related genes (Supplemental Table S2). PCR conditions were as follows: denaturation at 95 °C for 30 s and 41 cycles of 95 °C for 5 s and 60 °C for 34 s. The expression levels of *Actin* and *18* *S rRNA* used as internal standards. The comparative threshold method was used to calculate the relative mRNA levels. qRT-PCR experiments were performed in two independent trials with three replicatesin each test.

### GUS assays of TAL effector activity

To assay glucuronidase (GUS) reporter activity, *Agrobacterium tumefaciens* strains delivering TALEs and GUS reporter constructs were mixed 1:1 (OD_600_ = 0.8), and inoculated into 5–7 week old *Nicotiana benthamiana* leaves. For qualitative GUS assays, leaf discs were sampled 3 dpi, incubated in X-Gluc (5-bromo-4-chloro-3-indolyl-β-D-glucuronide)^30^, destained in ethanol, and dried on acetate foil sheets. For quantitative assays, two leaf discs (0.9 cm diameter) were sampled 2 dpi, and GUS activity was determined using 4-methyl-umbelliferyl-β-D-glucuronide (MUG). Proteins were quantified using Bradford assays. Data correspond to triplicate samples from different plants, and experiments were performed twice.

### Electrophoretic mobility shift assays (EMSA)

Promoter fragments 09g29100A (27 bp) and 09g29100B (32 bp) were amplified from rice cv. *Nipponbare* genomic DNA using EMSA primers EMSA9g-F2/EMSA9g-R2 and EMSA9g-F1/EMSA9g-R1, respectively (Supplemental Table S2). Promoter fragments 12g42970A (27 bp) and 12g42970B (30 bp) were bond using EMSA12g-F2/EMSA12g-R2 and EMSA12g-F1/EMSA12g-R1, respectively, in the binding buffer. The bond dsDNA sequences were labeled with the Biotin 3′ End DNA Labeling Kit (Thermo, USA). EMSA was performed using protocols supplied with the Light Shift Chemoluminescent EMSA Kit (Thermo).

Recombinant His-Tal7 was purified from *E*. *coli* BL21(DE3) containing pETtal7 (Supplemental Table S1). Bacteria were cultured in LB medium, induced, and harvested as described previously^9^. Cells were sonicated, centrifuged and fusion proteins were purified using Ni-NTA HisBind Resin (Novagen). 1 μl purified fusion proteins (20 μM His-Tal7) was mixed with 18 μl of binding buffer and 1 μl 20 fM of biotin-labeled DNA. The mixtures were incubated at room temperature for 30 min. Samples were then loaded on 5% polyacrylamide gels in 0.5 X TBE buffer, pH 8.3^59^. Gels were transferred to Hybond N^+^ membranes, and signals were detected by chemoluminescence according to the manufacturer’s instructions.

### Designer TAL effectors

TAL Effector-Nucleotide Targeter 2.0^43^ was used to design TAL effectors (dTALEs) that targeted 18-bp promoter regions in rice genes *Os09g29100* and *Os12g42970* (Supplemental Fig. 3). Four basic RVDs (NI, NG, NN, and HD) were used to recognize their respective target nucleotides (A, T, G and C) (Supplemental Fig. 3) described previously^63^. These RVDs were used to assemble the TAL effector central repeats based on the DNA sequence of the respective *Os09g29100* and *Os12g42970* promoter sites (dtal3-3 and dtal2-8, Supplemental Fig. 3). The central repeat regions of the dTALEs were synthesized by ViewSolid Biotechnology (Beijing, China) and cloned in pUAVPD^64^, resulting in pdTAL3-3 and pdTAL2-8 (Supplemental Table S1). These two plasmids were digested with *Sph* I to release the dTALEs and used to replace *avrXa7* in pZWavrXa7 at the *Sph* I site, resulting in pZWdtal3-3 and pZWdtal2-8 (Supplemental Table S1). The T3S signal, NLS and ADdomains of AvrXa7 were preserved in the two dTALE constructs (Supplemental Fig. 3c). Plasmids pZWdtal3-3 and pZWdtal2-8 were then linked to vector pHM1 at the *Hin*d III site, thus generating pHZWdtal3-3 and pHZWdtal2-8 (Supplemental Table S1); these constructs were transferred into *X*. *oryzae* and used in biological assays.

### Design and assembly of TALENs targeting the EBE_*tal7*_ in *Os09g29100*

We used TALENs to edit the EBE binding site in the promoter region of *Os09g29100*. The TALEN targeter program (https://tale-nt.cac.cornell.edu) was used to select two *Os09g29100* sequences as follows: left arm, 5′CCTACCCTCCACGCGGCT, and right arm, 5′GAGCAATGGGGGGAT (Supplemental Fig. 5b,c). The targeted TALENs were constructed by Sidansai Biotechnology (Shanghai, China) using the FastTALE^TM^ TALEN Assembly Kit and vectors pL20 and pR16, which contain *Fok*I DNA cleavage domains, Nos termini, and nuclear localization signals^65^. The TALEN backbone vectors pL20 and pR16 contain ubiquitin and 35S promoters, respectively (Supplemental Fig. 5b,c). The two TALEN expression cassettes were excised from pL20 and pR16 and ligated into the *Hin*d III *and Sac* I sites of plasmid pCAMBIA1301, resulting in pCAMBIA1301-EBE_tal7_ (Supplemental Fig. 5). The construct pCAMBIA1301-EBE_*tal7*_ was introduced into rice cv. *Nipponbare* by *Agrobacterium*-mediated transformation^66^, serviced by Wuhan Biorun Bio-Tech Co. Ltd. (Wuhan, China).

### Detection of mutations in the *Os09g29100* promoter

To detect and analyze mutations derived from TALEN editing, genomic DNA was extracted from T_0_ and T_1_ transgenic rice leaves and used for amplification of the *Os09g29100* promoter region with primers A97-F/R (Supplemental Table S2). The PCR products were subjected to T7 Endonuclease I (T7EI) assays to detect mismatched nucleotides as follows: PCR products (a mixture from mutant and wild-type) were heated at 95 °C for 5 min, then cooled from 95 to 75 °C at 0.05 °C s^−1^, 75 to 16 °C at 0.1 °C s^−1^, and then maintained at 16 °C for 2 min. The denatured and reannealed PCR products were digested with T7 endonuclease I (New England BioLabs) for 30 min at 37 °C; they were then subjected to 2% agarose gel electrophoresis. Mutant alleles were confirmed by subcloning and sequencing. *Nipponbare* and selected TALEN-edited Homozygous lines were inoculated with *Xoc* RS105 and YNB0-17 and virulence was assessed by phenotype and lesion length.

## Electronic supplementary material

Supplementary information
