# Supplementary material for: A Transcription Activator-Like Effector Tal7 of Xanthomonas oryzae pv. oryzicola Activates Rice Gene Os09g29100 to Suppress Rice Immunity
Source: Sci Rep. 2017 Jul 11;7:5089. doi: 10.1038/s41598-017-04800-8 (PMC5505973; doi:10.1038/s41598-017-04800-8)
Supplement: Supplementary file 1 — Supplementary information [file 41598_2017_4800_MOESM1_ESM.pdf]

SUPPORTING INFORMATION METHODS S1-S4, TABLE S1 AND FINGURES S1-S6

Article title: A Transcription Activator-Like Effector Tal7 of *Xanthomonas oryzae* pv. *oryzicola* Activates Rice Gene *Os09g29100* to Suppress Rice Immunity

Authors: Lulu Cai, Yanyan Cao, Zhengyin Xu, Wenxiu Ma, Muhammad Zakria, Lifang Zou, Zaiquan Cheng*, & Gongyou Chen*

Supporting information Methods S1-S4:

Methods S1: Genomic DNA extraction and Southern blot analysis

Genomic DNAs were extracted from *X. oryzae* strains using the AxyPrep Bacterial Genomic DNA Miniprep Kit (Axygen, Hanzhou, China) and digested with *Bam*HI and *Sph*I. The digested genomic DNA fragments were fractionated on a 1.2% agarose gel and then transferred to Amersham Hybond^{TM}-N+ membranes (GE Healthcare, Buckinghamshire, USA). *avrXa10* was digested with *Bam*HI, and a 3.1-kb fragment containing the repeat region was extracted and labeled with digoxigenin (DIG) High Prime (Roche, Sweden). Standard procedures for labeling, hybridization and detection were utilized as recommended (Roche, Sweden).

Methods S2: RT-PCR

Strains were inoculated to 20 ml NB and incubated at 28 ℃ for 24-36 h until the OD_{600} value reached 0.8; two milliliters of this culture was then transferred into 100 ml of fresh NB and incubated another 12-16 h until the OD_{600} = 0.6. After centrifugation at 6,000 rpm for 10 min at 4 ℃, the pellet was washed twice in sterile distilled water with centrifugation for 10 min. The pellet was then suspended in sterile water to OD_{600} = 2.0, and 40 μl of this bacterial suspension[1] was incubated with one milliliter of rice suspension cells. After incubation for 16 h at 26℃, bacteria were collected by centrifugation at 12,000 rpm for 5 min, and total RNA was extracted using Trizol reagent according to the manufacturer's instructions (Invitrogen, Carlsbad, USA). The RNA was quantified by measuring the OD_{260}/OD_{280}, and the quality was

inspected by gel electrophoresis. Before first strand synthesis, RNA was digested with RNase-free DNase I (TaKaRa, Dalian, China) to remove potential traces of genomic DNA and then used as a template to amplify selected genes using the primers listed in Table S1. cDNA synthesis and PCR were accomplished using AMV and Ex-Taq DNA polymerase (TaKaRa), respectively. PCR was performed with a thermal cycler using the following parameters: 94°C for 35 s, 52°C for 30 s, and 72°C for 30 s. The amplification products were analyzed in 1.2% agarose gels. The *16S* rRNA gene of *X. oryzae* was used as an internal control to normalize cDNA loading levels.

Methods S3: Affinity measurements by microscale thermophoresis (MST)

In addition to EMSA, microscale thermophoresis (MST) was used to evaluate the interaction of Tal7 and AvrXa7 with the promoter regions of three rice genes (*Os09g29100*, *Os12g42970*, and *Os11N3*). MST was performed using a Monolith NT.115 (NanoTemper Technologies GmbH, Munich, Germany). LED power was set at 20, 40 or 80% and IR laser power was 40 or 80%. The promoter regions of the three rice genes were located 200 bp upstream from the translational start codon and were amplified from rice *Nipponbare* genomic DNA using primers sets 09g-F/09g-R,12g-F/12g-R and 11N3-F/11N3-R (Supplemental Table S2). Primers 09g-F, 12g-F and 11N3-F were synthesized and 5'-FAM-labeled by Generay Biotechnology Co. (Shanghai, China). A series of 16 AvrXa7 and Tal7 protein solutions were prepared by consecutive 2-fold dilution from an initial concentration of 10 µM. Protein solutions and dsDNA (20 fM) were mixed 1:1 v/v, incubated at room temperature for 20 min, and loaded into silica capillaries. Measurements were performed at 25°C in MST optimized buffer [50 mM Tris (pH 7.4) with 150 mM NaCl, 10 mM $MgCl_2$ and 0.05% (v/v) Tween-20]. Dissociation contants (K_d) were calculated using MO. Affinity Analysis Software (NanoTemper Technologies GmbH). Data were analysed using Origin v. 8.5 software (www.originlab.com).

Methods S4: Construction *avrXa7* together with *avrRxo1* or *xopO*

To express *avrXa7* together with *avrRxo1* or *xopO*, the coding regions with native promoters of *avrRxo1* or *XopO* were amplified from strain RS105 with the primers avrRxo1-orfF/avrRxo1-orfF or xopO-orfF/xopO-orfR (Supplemental Table S2) containing a

*Sac*I site at the 5' and 3' termini. After sequence verification, the fragments were cloned to pAvrXa7 at *Sac*I site, generating the chimeric constructs pavrXa7A and pavrXa7X (Supplemental Table 1), where A and X represent *avrRxo1* and *XopO*, respectively. These constructs were then transformed into strains YNB0-17 and RS105 by electroporation with a Gene Pulser II according to the manufacturer's protocol (Bio-Rad, USA).

Supporting information Table S1-S2:

Table S1 Bacterial strains and plasmids used in this study.

Strain or plasmid	Relevant characteristics[a]	Reference or source
E. coli		
DH5α	F^- *endA1*, *thi-1*, *recA1*, Φ80*lacZ*, ΔM15	This lab
BL21(DE3)	F^- *ompT, hsdS20, gal*	This lab
X. oryzae pv. *oryzicola*		
JSB2-24	Wild-type isolate from Jiangsu province containing a larger number of *tale* genes than strain RS105	This lab
RS105	Dominant strain causing BLS in China; contains over 20 *tale* genes, Rif^r	Zou *et al*.[37]
YNB0-17	Hypovirulent isolate from Yunnan province; contains nine *tale* genes	This study
RΔ*hrcV*	RS105 containing a deletion in *hrcV*, Rif^r	This lab
X. oryzae pv. oryzae		
$PXO99^A$	Wild-type, Philippine race 6, 5-azacytidine resistant; Rif^r	This lab
PH	Derived from $PXO99^A$; *tale*-free strain; Rif^r	This lab
PXO86	Wild-type, Philippine race 2	This lab
A. tumefaciens		
EHA105	Rif^r	This lab
Plasmids		
pHM1	Broad-spectrum cosmid vector, *cos*, *parA*, *IncW*	This lab
pUFR034	*IncW*, *Mob(p), Mob+, LacZa*+, PK2 replicon, cosmid, Km^r	This lab
pZWavrXa7	*avrXa7* in pBluescript II KS +, Ap^r	Yang *et al*.[24]
pZWdtal3-3	Contains the central repeat region designed to target an 18-kb region in the *Os09g29100* promoter; replaced *avrXa7* in pZWavrXa7 at the *SphI* site, Ap^r	This study
pZWdtal2-8	Contains the central repeat region designed to target an 18-kb region in the *Os12g42970* promoter; replaced *avrXa7* in pZWavrXa7 at the *SphI* site, Ap^r	This study
pHZWavrXa7	pHM1 carrying *avrXa7* fused with *lacZ* promoter, Sp^r	Yang *et al*.[24]
pHZWavrXa10	pHM1 carrying *avrXa10* fused with *lacZ* promoter, Sp^r	Yang *et al*.[24]
pHZWavrXa27	pHM1 carrying *avrXa27* fused with *lacZ* promoter, Sp^r	Gu *et al*.[32]
pHZWtal7	pHM1 carrying *tal7* fused with *lacZ* promoter, Sp^r	This study
pHZWdtal3-3	pHM1 carrying *dtal3-3* fused with *lacZ* promoter, Sp^r	This study
pHZWdtal2-8	pHM1 carrying *dtal2-8* fused with *lacZ* promoter, Sp^r	This study

pavrXa7	pUFR034 carrying *avrXa7* under a *tale* conserved promoter, Km^r	This study
ptal7	pUFR034 carrying *tal7* under a *tale* conserved promoter, Km^r	This study
pavrXa7A	pUFR034 expressing *avrXa7* and *avrRxo1* under their native promoters, Km^r	This study
pavrXa7X	pUFR034 expressing *avrXa7* and *xopO* under their native promoters, Km^r	This study
p707	pUFR034 expressing *avrXa7*-His and *tal7*-c-Myc under their native promoters, Km^r	This study
p707ΔNA	pUFR034 expressing *avrXa7*-His and *tal7ΔNA* – c-Myc under their native promoters, Km^r	This study
pET30a (+)	pBR322 origin, F1 origin, *lacI*, His-Tag, S-Tag, Km^r	Novagen
pETtal7	pET30a expressing *tal7* with N-terminal His-tag, Km^r	This study
pETavrXa7	pET30a expressing *avrXa7* with N-terminal His-tag, Km^r	This study
PHB	pUC *oriV*, binary vector for transient expression, Ap^r Km^r	This lab
PHB-tal7	PHB containing *tal7*, Ap^r	This study
PHB-avrXa7	PHB containing *avrXa7*, Ap^r	This study
PHB-pthXo1	PHB containing *pthXo1*, Ap^r	This study
pCAMBIA1381	Binary vector for transient expression, Km^r	This lab
p09g29100	pCAMBIA1381 containing promoter of *Os09g29100* fused to *gusA*, Km^r	This study
p12g42970	pCAMBIA-1381 containing promoter of *Os12g42970* fused to *gusA*, Km^r	This study
pOs8N3	pCAMBIA1381 containing *Os8N3* promoter fused to *gusA*, Km^r	This study
pUAVPD	Derived from pUFR034 and pUAV45, contains *avrXa3*, Sp^r, Km^r	Wu *et al*.[64]
pdTAL3-3	pUAVPD containing a central repeat region designed to target an 18-kb region in the *Os09g29100* promoter, Sp^r	This study
pdTAL2-8	pUAVPD containing a central repeat region designed to target an 18-kb region in the *Os12g42970* promoter, Sp^r	This study

[a]Rif^r, Sp^r, Ap^r and Km^r indicate resistance to rifampicin, spectinomycin, ampicillin, and kanamycin, respectively.

Table S2 Primers used in this study

Primers	Sequence (5'→3')	Description of fragment amplified
16s rRNA-F	TTAGAGTTCCCACCATTACGT	800-bp fragment of *16S rRNA*
16s rRNA-R	CAGCAGTGGGGAATATTGGAC	
hrcC-F	ATGCAGAGCTATGGCACCCT	567-bp fragment of *hrcC*
hrcC-R	GCTGGAGGTAATGACCGGAAT	
hpa1-F	TTCCAGGTTGACCAAAGCCA	346-bp fragment of *hpa1*
hpa1-R	AGAAGCTGCTGGCGAAACTG	
hpa3-F	TAGTGCAGCAGCCACCTCTTCA	357-bp fragment of *hpa3*
hpa3-R	GGAATCGCAGAATCAAACAGCA	
avrRxo1-F	GCATCCCGAGCGCCGTAAGTTACACG	408-bp fragment of *avrRxo1*
avrRxo1-R	CTATCAATTTCGTACTCTCTTGACAA	
XopO-F	TTAATGGCCATCATATCCACTCCTCT	636-bp fragment of *xopO*
XopO-R	ATACACCACGACTTGCCCCATCAAAG	
avrRxo1-orfF	ATAGAGCTCGATCGCAGCATGCGACGCATTTTTATAGC	1473-bp fragment containing *avrRxo1* and its promoter
avrRxo1-orfR	TATGAGCTCAGCTTCTTATGAAATTCGAGTGCGTCTGC	
XopO-orfF	ATAGAGCTCGCTGTCGGAATGGATCGGGCAACGCCTGC	1391-bp fragment containing x*opO* and its promoter
XopO-orfR	TATGAGCTCCGGATTAGCGCCGGTCTCCTTGGCGACCA	
tal-pF	TGGGTACCATCTACCTGCGGGACGTAC	280-bp containing a conserved *tal* promoter region upstream of the translational start
tal-pR	CCAGGATCCATCAGGCATACCTCTTTA	
tal-tF	GCCTGATGGATCCTGGTACGCCCATCG	210-bp fragment containing a conserved region downstream of the *tal* translational stop
tal-tR	TTGGGTACCGTTTCTTACTCGAATCCC	
tal-KHKF	ATAGGTACCGTGATGACCTCGCTGGCCTTC	880-bp of *pthXo1* C-terminal region in strain PXO99[A] (nucleotides: 1644538-1645417)
tal-KHKR	CCCGGATCCTGGTACGCCCATCGCTGCCGA	

tal-KHHF<br>*tal*-KHHR	TGGGGTCCGGGCAGAAGCTCGCGGGCAGG<br>ATTCGAGTAAGAAAGCTTTACTGACAGCAA	1036-bp of *pthXo1* promoter region in strain PXO99[A] (nucleotides: 1649303-1650338)
tal-HKHF<br>*tal*-HKHR	CTGTCAGTAAAGCTTTCTTACTCGAATCCC<br>CGGGGGCGGCCTCCCGGATCCTGGTACGCC	229-bp of *tal2a* C-terminal region in strain PXO99[A] (nucleotides: 1650312-1650540)
tal-HKKF<br>*tal*-HKKR	ACGAATGGGATCCATCAGGCATACCTCTTT<br>TATGGTACCGTGCGCAAACGTGCGCGCATC	990-bp of *tal2a* promoter region in strain PXO99[A] (nucleotides: 1653543-1654532)
*Xa27*F<br>*Xa27*R	CAAGAAGCTGCCTCCAATGGCGGATTG<br>TCCGCCGCCGGTAGTGGTGAGCATGTG	188-bp fragment of *Xa27*
*Os11N3*F<br>*Os11N3*R	TTCTCCTTCGGCGTCATCCAGATGGGG<br>GCCGGCGGGGACTCGACGTCGACGGGG	225-bp fragment of *Os11N3*
Actin-F<br>*Actin-R*	GAAGATCACTGCCTTGCTCC<br>CGATAACAGCTCCTCTTGGC	120-bp fragment of *Actin* gene
18S rRNA-F<br>*18S rRNA-F*	ATGATAACTCGACGGATCGC<br>CTTGGATGTGGTAGCCGTTT	132-bp fragment of *18S rRNA*
Q01g-F<br>*Q01g-R*	TTTTGGCCTTTGGTTTGTGG<br>AGTATTTGGCATGGCCTGAC	109-bp fragment of *Os01g31220*
QO7g-F<br>*Q07g-R*	CCCCCGACCAAGGTAGTAGC<br>GCCGCTACAGACACGCATAC	110-bp fragment of *Os07g47790*
Q02g-F<br>*Q02g-R*	GGTATCCGAGGACGACAAGC<br>CTCGTCCCTGTCGTTCTCGT	151-bp fragment of *Os02g14770*
Q09g-F<br>*Q09g-R*	CACATGCCGAGGGAGGACTA<br>GCAGGCAGTAAGAGGAGCGA	141-bp fragment of *Os09g29100*
Q12g-F<br>*Q12g-R*	AGGAGCCTGGCATCTGTCAT<br>ATCGAGCAGGAGTAGCGGTG	84-bp fragment of *Os12g42970*
EMSA9g-F1<br>*EMSA9g-R1*	CCACACAATCTATCACAAGCTTAGCAAGATTT<br>AAATCTTGCTAAGCTTGTGATAGATTGTGTGG	32-bp fragment of *Os09g29100* promoter region

EMSA9g-F2<br>*EMSA9g-R2*	GTATACAAAGAGAACGCATCCCCCCAT<br>ATGGGGGGATGCGTTCTCTTTGTATAC	27-bp fragment of *Os09g29100* promoter region
EMSA12g-F1<br>*EMSA12g-R1*	ACCTACCCCCTCTTCTTCCTCCGCTTCTCT<br>AGAGAAGCGGAGGAAGAAGAGGGGGTAGGT	30-bp fragment of *Os12g42970* promoter region
EMSA12g-F2<br>*EMSA12g-R2*	AGATAAAAGCGAAACCCTCCCTCCCCT<br>AGGGGAGGGAGGGTTTCGCTTTTATCT	27-bp fragment of *Os12g42970* promoter region
MST-9g-F<br>*MST-9g-R*	CTGCCCAAAATGCCCTCCTC<br>TTCCTTGCTGATAAATCTTGCTAAG	200-bp fragment of *Os09g29100* promoter region
MST-12g-F<br>*MST-12g-R*	CTTGCTCGGCTCGGCTCACG<br>GCCCAGCGACGAGGAGGAGGCAGCC	200-bp fragment of *Os12g42970* promoter region
MST-Os11N3-F<br>*MST-Os11N3-R*	TATATTGCCTATTGGTGTCCA<br>TAATCAGCTACTACTTGAGT	100-bp fragment of *Os11N3* promoter region
A97-F<br>*A97-R*	GCAACTAATGCTCAGACTCC<br>CCGCCATCTCGTAGCTCGTC	Amplifies an 443-bp region from the promoter of *Os09g29100*

Supporting information Figures S1-S6:

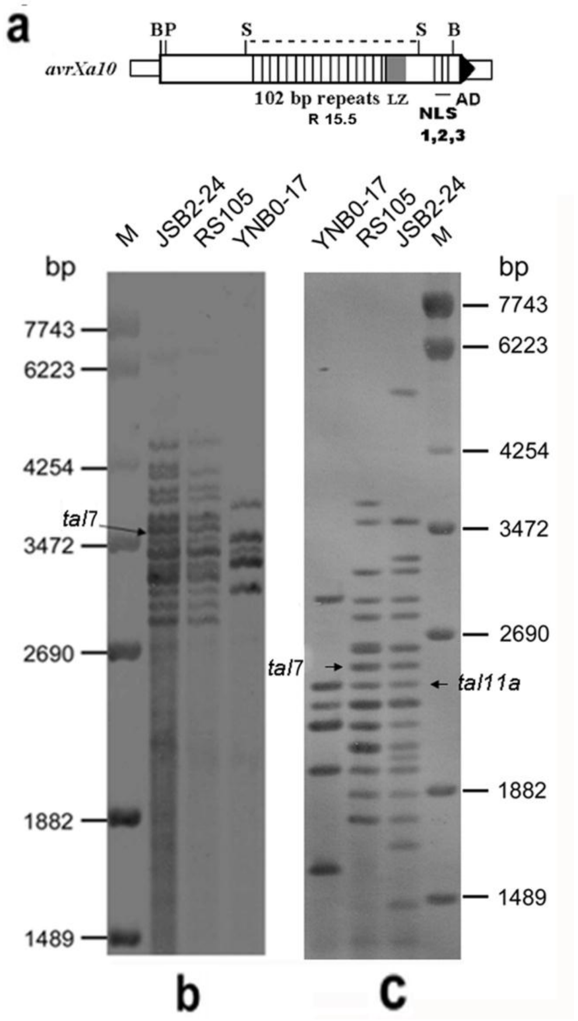

Supplemental Figure 1. Identification of putative *tal* genes in *X. oryzae* pv. *oryzicola* (*Xoc*) strains. (a) Diagrammatic representation of the *tal* gene *avrXa10*. Abbreviations: B, P and S represent *Bam*HI, *Pst*I and *Sph*I sites; R15.5 indicates the number of repeats of a 102-bp unit; NLS, nuclear localization signals; AD, acidic transcriptional activation domain; and LZ, leucine zipper region. The dashed line above the map displays the probe used for Southern blot analysis (see Methods S1). Panels (b) and (c) show the numbers of *tal* genes detected in *Xoc* strains JSB2-24, RS105 and YNB0-17. Genomic DNAs of the tested strains were digested with *Bam*HI (panel b) or *Sph*I (panel c), blotted to nylon membranes, and hybridized with the *Sph*I fragment from *avrXa10*. Black arrows indicate the location of *tal7* in hybridizing DNA. M, λ-*Eco*T14 (TaKaRa, Dalian, China).

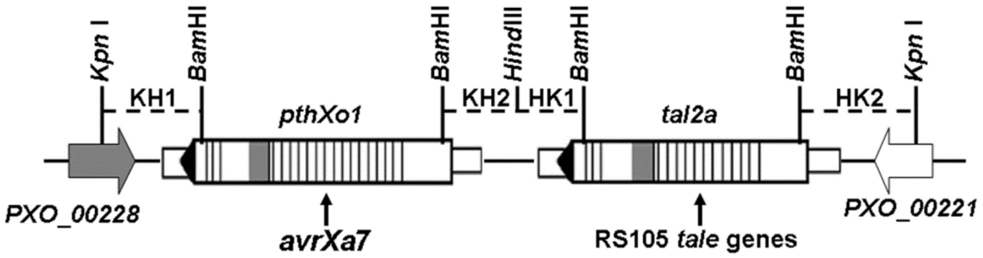

Supplemental Figure 2. Methods used for introducing *avrXa7* and candidate *tal* genes into *Xoc* RS105. Primers *tal*-KHKF/*tal*-KHKR (producing KH1), *tal*-KHHF/*tal*-KHHR (KH2), *tal*-HKHF/*tal*-HKHR (HK1) and *tal*-HKKF/*tal*-HKKR (HK2) (Table S1) were synthesized to amplify the upstream (KH2 and HK2) and downstream (KH1 and HK1) fragments of two *tal* genes (*pthXo1* and *tal2a*) in cluster 2 of PXO99^{A} [60]. A unique *Hin*dIII site was created using single nucleotide mutation (G→C) in the primer pairs. The upstream fragments KH1 and KH2 were cloned into pMD18-T and integrated with the corresponding downstream fragments (HK1 and HK2) to create the basic construct. The central *Bam*HI fragments of *avrXa7* and candidate *tal* genes derived from *Xoc* RS105 were inserted into *pthXo1* and *tal2a* (indicated by vertical arrows), respectively. The resulting *tal* cluster was cloned into the vector pUFR034 at the *Kpn*I site. For Western blots, *avrXa7* with a C-terminal 3X-Flag and a *tal* (or truncated *tal*) from strain RS105 with a C-terminal 3X c-Myc tag were constructed.

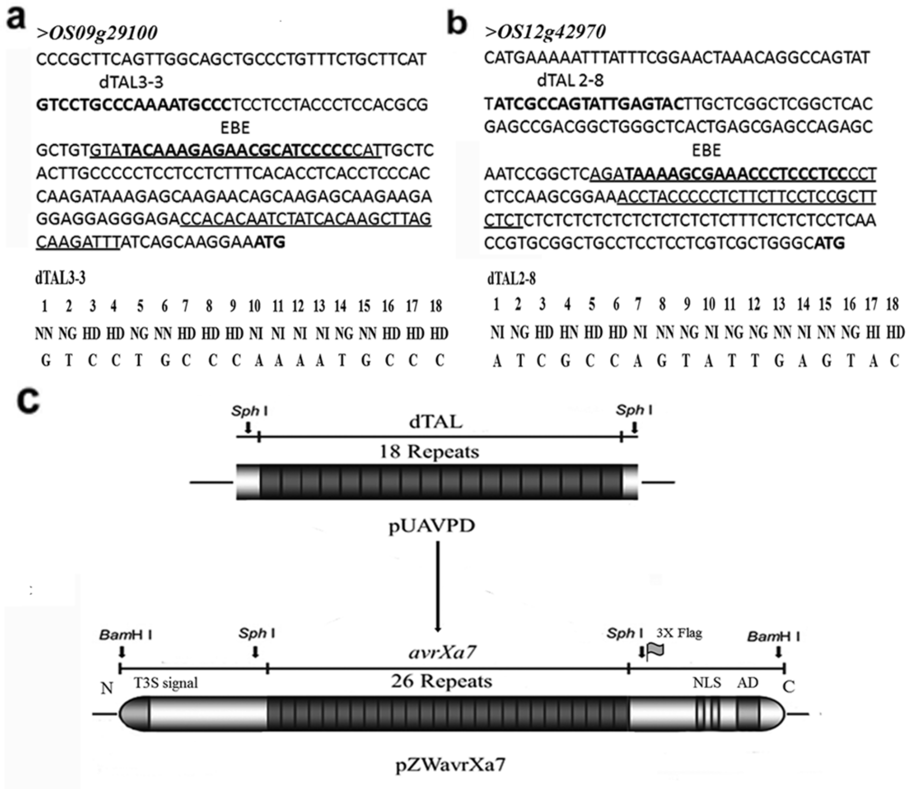

Supplemental Figure 3. The promoters of *Os09g29100* and *Os12g42970*, and the dTAL effectors dTAL3-3 and dTAL2-8 were designed based on the promoter regions of the two rice genes. (a) Schematic of the *Os09g29100* promoter; dTAL3-3 was designed to target an 18-bp region upstream of the endogenous effector site (EBE_{tal7}). The 18 repeat variable diresidues (RVDs) in dTAL3-3 and their nucleotide targets are shown. (b) Schematic of the *Os12g42970* promoter; dTAL2-8 was designed to target an 18-bp region upstream of the EBE_{tal7}. The 18 RVDs in dTAL2-8 and their nucleotide targets are shown. In (a) and (b)，translational start sites are shown in boldface (ATG) as predicted by the Rice Genome Annotation Project (http://rice.plantbiology.msu.edu). The effector binding elements (EBEs) for Tal7 are underscored, and the two underscored regions in each promoter were used in EMSA. The 18-bp sequences used to design custom TAL effectors are shown in boldface and labeled dTAL3-3 and dTAL2-8, which target *Os09g29100* and *Os12g42970*, respectively. (c) Functional map showing the insertion of the dTAL RVDs into the *Sph*I fragment containing the RVDs in AvrXa7. This event deleted the central repeats in AvrXa7, but maintained the endogenous T3S signal, NLS, and AD domains.

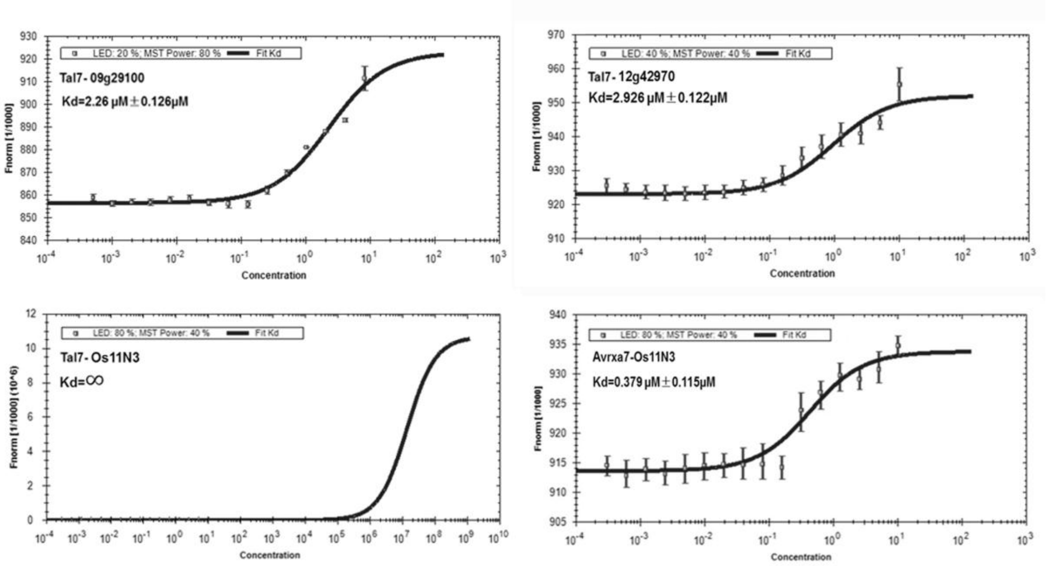

Supplemental Figure 4. The affinity of effector Tal7 for three rice promoter regions using microscale thermophoresis (MST). The promoter regions of rice genes *Os09g29100*, *Os12g42970*, and *Os11N3* were located 200 bp upstream from the translational start codon. These regions were amplified from genomic DNA and 5'-FAM-labeled (see Methods S2). A dilution series of 16 Tal7 protein solutions were prepared by consecutive 2-fold dilutions from an initial concentration of 10 μM. Protein solutions and dsDNA (20 fM) were mixed 1:1 v/v, incubated at room temperature for 20 min, and loaded into silica capillaries. Measurements were performed at 25°C in MST optimized buffer. Comparisons were made using 20, 40 or 80% LED power and 40 or 80% IR-laser power. Data were analyzed using Origin v. 8.5, and each experiment was repeated at least three times.

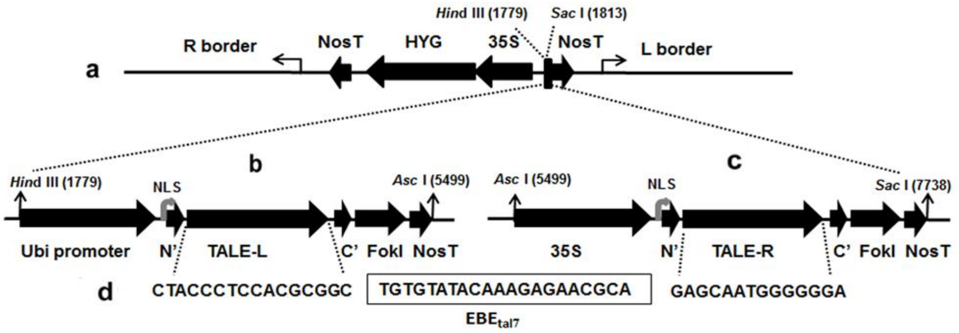

e **TALE-L**

LTPEQVVAIASHDGGKQALETVQRLLPVLCQAHGLTPEQVVAIASHDGGKQALETVQRLLPVLCQAHGLTPEQVVAIASNGGG
KQALETVQRLLPVLCQAHGLTPEQVVAIASNIGGKQALETVQRLLPVLCQAHGLTPEQVVAIASHDGGKQALETVQRLLPVLCQ
AHGLTPEQVVAIASHDGGKQALETVQRLLPVLCQAHGLTPEQVVAIASHDGGKQALETVQRLLPVLCQAHGLTPEQVVAIASN
GGGKQALETVQRLLPVLCQAHGLTPEQVVAIASHDGGKQALETVQRLLPVLCQAHGLTPEQVVAIASHDGGKQALETVQRLLP
VLCQAHGLTPEQVVAIASNIGGKQALETVQRLLPVLCQAHGLTPEQVVAIASHDGGKQALETVQRLLPVLCQAHGLTPEQVVAI
ASNNGGKQALETVQRLLPVLCQAHGLTPEQVVAIASHDGGKQALETVQRLLPVLCQAHGLTPEQVVAIASNNGGKQALETVQ
RLLPVLCQAHGLTPEQVVAIASNNGGKQALETVQRLLPVLCQAHGLTPEQVVAIASHDGGKQALETVQRLLPVLCQAHG

f **TALE-R**

LTPEQVVAIASNNGGKQALETVQRLLPVLCQAHGLTPEQVVAIASNIGGKQALETVQRLLPVLCQAHGLTPEQVVAIASNNGGK
QALETVQRLLPVLCQAHGLTPEQVVAIASHDGGKQALETVQRLLPVLCQAHGLTPEQVVAIASNIGGKQALETVQRLLPVLCQA
HGLTPEQVVAIASNIGGKQALETVQRLLPVLCQAHGLTPEQVVAIASNGGGKQALETVQRLLPVLCQAHGLTPEQVVAIASNNG
GKQALETVQRLLPVLCQAHGLTPEQVVAIASNNGGKQALETVQRLLPVLCQAHGLTPEQVVAIASNNGGKQALETVQRLLPVL
CQAHGLTPEQVVAIASNNGGKQALETVQRLLPVLCQAHGLTPEQVVAIASNNGGKQALETVQRLLPVLCQAHGLTPEQVVAIA
SNNGGKQALETVQRLLPVLCQAHGLTPEQVVAIASNIGGKQALETVQRLLPVLCQAHG

Supplemental Figure 5. TALEN sequences and vector construction. (a) Functional map of pCAMBIA1301M; the vector used for *Agrobacterium*-mediated plant transformation. (b) Left-arm cassette containing TALE-L in vector pL20. (c) Right-arm cassette containing TALE-R in vector pR16. The TALEN expression cassettes were excised with *Hin*dIII/*Asc*I (left-arm cassette in pL20) and *Asc*I/*Sac*I (right-arm cassette in pR16) and cloned into the *Hin*dIII/*Sac*I site of pCAMBIA1301M, resulting in pCAMBIA1301-EBE$_{tal7}$. (d) The discrimination sequences recognized by TALE-L and TALE-R and the target site (bordered with rectangle) in the promoter region of *Os09g29100*. (e) Amino acid sequence of TALE-L, which recognizes CTACCCTCCACGCGGC in the promoter of *Os09g29100*. (f) Amino acid sequence of TALE-R, which recognizes the target GAGCAATGGGGGGAT in *Os09g29100*. The sequences of pL20 and pR16 were recently published by Wang et al[2]. Abbreviations: NosT, nopaline synthase termination sequence; HYG, hygromycin resistance; Ubi, ubiquitin promoter sequence; 35S, promoter sequence from CaMV.

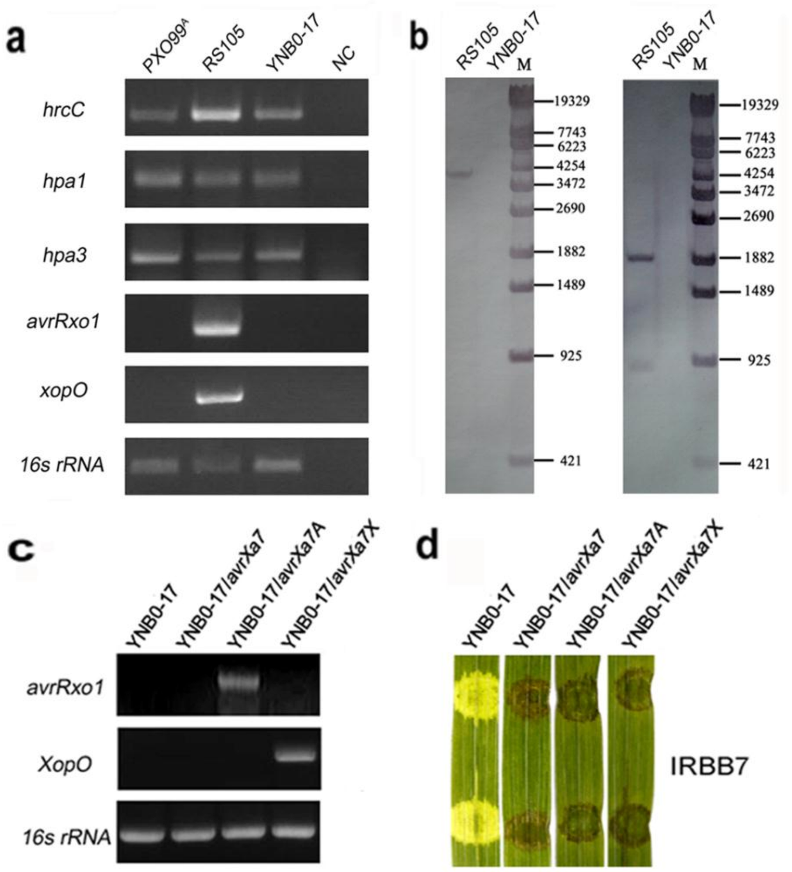

Supplemental Figure 6. YNB0-17 contains the T3SS genes but lacks the effectors AvrRxo1 and XopO, and that two NTALEs do not impact ETI in this strain. (a) Semiquantitative RT-PCR analysis of representative T3SS genes (*hrcC*, *hpa1*, and *hpa3*) and two *ntal* genes (*avrRxo1* and *xopO*) in *Xoc* strains RS105 and YNB0-17. The wild-type *Xoo* strain PXO99^{A} was used for comparative purposes. NC, represents a negative control using the extracted RNA as the template. The *16S rRNA* gene was used as an internal control to verify the absence of significant variation in cDNA levels. (b) *Xoc* strains YNB0-17 and RS105 were digested with *Eco*RI and hybridized with 408- or 637-bp PCR products representing *avrRxo1* and *xopO*, respectively (Supplemental Table S2). Hybridization of *Eco*RI-digested genomic DNA of *Xoc* strains YNB0-17 and RS105 with the 408-bp *avrRxo1* PCR product (left lane) and 638-bp *xopO* PCR product (right lane). M, Molecular weight markers derived from λ-*Eco*T14; numbers indicate the size in base pairs. (c) Expression of *avrRxo1* and *xopO* in *Xoc* strain YNB0-17. The *avrRxo1* and *xopO* genes were cloned in pavrXa7, producing pavrXa7A and pavrXa7X. Strains containing pUFR034 (empty vector, lane 1) or *avrXa7* (lane 2) were used as negative controls. The *16S rRNA* gene was used as an internal control to verify the cDNA quantity in

samples. (d) Co-expression of *avrRxo1* or *xopO* with *avrXa7* does not inhibit *avrXa7-Xa7* mediated ETI in *Xoc* YNB0-17. *Xoc* strains containing the plasmid constructs were inoculated to seedlings of rice line IRBB7. Water-soaked symptoms indicate susceptibility to *Xoc* and dark-brown necrotic regions represent an HR and resistance to *Xoc*. Photos were taken 3 dpi. The experiment was repeated twice with similar results.

Supplementary References

1. Li, Y. R. et al. Hpa2 required by HrpF to translocate Xanthomonas oryzae transcriptional activator-like effectors into rice for pathogenicity. App Environ Microb. 77, 3809–3818 (2011).

2. Wang, M. et al. Gene editing by co-transformation of TALEN and chimeric RNA/DNA oligonucleotides on the rice OsEPSPS gene and the inheritance of mutations. PLoS One. 10, e0122755 (2015).
